# Control of *CDH1*/E-Cadherin Gene Expression and Release of a Soluble Form of E-Cadherin in SARS-CoV-2 Infected Caco-2 Intestinal Cells: Physiopathological Consequences for the Intestinal Forms of COVID-19

**DOI:** 10.3389/fcimb.2022.798767

**Published:** 2022-05-04

**Authors:** Ikram Omar Osman, Clémence Garrec, Gabriel Augusto Pires de Souza, Ana Zarubica, Djamal Brahim Belhaouari, Jean-Pierre Baudoin, Hubert Lepidi, Jean-Louis Mege, Bernard Malissen, Bernard La Scola, Christian Albert Devaux

**Affiliations:** ^1^ Microbes Evolution Phylogeny and Infections (MEPHI), Institut de recherche pour le Développement (IRD), Assistance Publique Hôpitaux de Marseille (APHM), Institut Hospitalo-Universitaire (IHU)-Méditerranée Infection, Marseille, France; ^2^ Aix-Marseille Université, Marseille, France; ^3^ Centre d’Immunophénomique (CIPHE), Aix Marseille Université, Institut National de la Santé et de la Recherche Médicale (INSERM), Centre National de la Recherche Scientifique (CNRS), CELPHEDIA, PHENOMIN, Marseille, France; ^4^ Assitance Publique Hôpitaux de Marseille (APHM), Marseille, France; ^5^ Centre National de la Recherche Scientifique (CNRS), Marseille, France

**Keywords:** SARS-CoV-2, E-cadherin, gastrointestinal tract, COVID-19, infection, intestinal barrier

## Abstract

COVID-19 is the biggest pandemic the world has seen this century. Alongside the respiratory damage observed in patients with severe forms of the disease, gastrointestinal symptoms have been frequently reported. These symptoms (e.g., diarrhoea), sometimes precede the development of respiratory tract illnesses, as if the digestive tract was a major target during early SARS-CoV-2 dissemination. We hypothesize that in patients carrying intestinal SARS-CoV-2, the virus may trigger epithelial barrier damage through the disruption of E-cadherin (E-cad) adherens junctions, thereby contributing to the overall gastrointestinal symptoms of COVID-19. Here, we use an intestinal Caco-2 cell line of human origin which expresses the viral receptor/co-receptor as well as the membrane anchored cell surface adhesion protein E-cad to investigate the expression of E-cad after exposure to SARS-CoV-2. We found that the expression of *CDH1*/E-cad mRNA was significantly lower in cells infected with SARS-CoV-2 at 24 hours post-infection, compared to virus-free Caco-2 cells. The viral receptor ACE2 mRNA expression was specifically down-regulated in SARS-CoV-2-infected Caco-2 cells, while it remained stable in HCoV-OC43-infected Caco-2 cells, a virus which uses HLA class I instead of ACE2 to enter cells. It is worth noting that SARS-CoV-2 induces lower transcription of TMPRSS2 (involved in viral entry) and higher expression of B^0^AT1 mRNA (that encodes a protein known to co-express with ACE2 on intestinal cells). At 48 hours post-exposure to the virus, we also detected a small but significant increase of soluble E-cad protein (sE-cad) in the culture supernatant of SARS-CoV-2-infected Caco-2 cells. The increase of sE-cad release was also found in the intestinal HT29 cell line when infected by SARS-CoV-2. Beside the dysregulation of E-cad, SARS-CoV-2 infection of Caco-2 cells also leads to the dysregulation of other cell adhesion proteins (occludin, JAMA-A, zonulin, connexin-43 and PECAM-1). Taken together, these results shed light on the fact that infection of Caco-2 cells with SARS-CoV-2 affects tight-, adherens-, and gap-junctions. Moreover, intestinal tissues damage was associated to the intranasal SARS-CoV-2 infection in human ACE2 transgenic mice.

## Introduction

Since its first description in China in 2019, the coronavirus disease 2019 (COVID-19) has emerged as a world pandemic. Insomuch its aetiological agent, SARS-CoV-2, was considered to be an exclusive airborne pathogen with a preferential tropism for the angiotensin converting enzyme 2 (ACE2)-positive epithelial cells of the pulmonary alveoli (Devaux et al., 2020; Huang et al., 2020; Yan et al., 2020; Zhu et al, 2020), other routes of infection (e.g., oral route) and tropism for other tissues (e.g., intestinal epithelium) received less attention, despite growing importance in the pathophysiology of COVID-19 (Lamers et al., 2020; Xiao et al., 2020a; Devaux et al., 2021). Gastrointestinal tract (GIT) symptoms, including diarrhoea, nausea, abdominal pain, and vomiting, have been frequently reported in COVID-19 patients (D’Amico et al., 2020; Lin et al., 2020; Song et al., 2020; Wang et al., 2020; Zhang et al., 2020a). Moreover, GIT-symptoms sometimes precede the development of respiratory tract symptoms (Fang et al., 2020; Li et al., 2020; Pan et al., 2020; Redd et al., 2020), and people with GIT-symptoms were much more likely to have the SARS-CoV-2 detected in their stool samples (Han et al., 2020; Wölfel et al., 2020; Xu et al., 2020; Zheng et al., 2020). The process by which SARS-CoV-2 reaches the intestine is not yet clear and could occur either by the bloodstream (with or without a hepatic stage) or by the oral-intestinal route (from the trachea to the esophagus and intestine). The potential role of the oral-intestinal transmission of SARS-CoV-2 is currently considered as likely (Brogna et al., 2022; Heneghan et al., 2021; Meng and Liang, 2021; Wendling et al., 2021; Dergham et al., 2021). SARS-CoV-2 replication in the GIT is associated with modulation in the diversity of bacterial species (Dhar and Mohanty, 2020; Gu S. et al., 2020; Gu J. et al., 2020; Zuo et al., 2020). As in many invasive infection processes initiated within the GIT, the pathogen is expected to develop strategies aimed at destroying the adherens junctions insured by cell adhesion molecules (CAM), such as E-cadherin (E-Cad), to create epithelium micro-damage, to dysregulate the immune response, and/or to invade the host (Devaux et al., 2019). Several viruses with intestinal tropism such as Hepatitis B virus and Hepatitis C virus, and viruses with other organ tropism, modulate E-cad expression (Lee et al., 2005; D'Costa et al., 2012; Li et al., 2016). Using a mouse model expressing transgenic human ACE2 it was demonstrated that intragastric inoculation of SARS-CoV-2 causes productive infection, virus shedding was found in faeces, and the viral invasion *via* the GIT lead to secondary pulmonary pathological changes (Sun et al., 2021). Similar observations were reported using nonhuman primates as a model of SARS-CoV-2 intestinal infection (Jiao et al., 2021).

SARS-CoV-2 was reportedly able to infect human small intestinal organoids established from primary gut epithelial stem cells (Lamers et al., 2020). Intestinal biopsies of COVID-19 patients evidenced the presence of replicating SARS-CoV-2 in epithelial cells of the small and large intestine (Xiao et al., 2020b). The main SARS-CoV-2 receptor, ACE2, was found in the small intestine with the highest expression observed in the brush border of intestinal enterocytes (Hamming et al., 2004; Qi et al., 2020; Zuo et al., 2020). Although the most well-known physiological function of ACE2 is the regulation of the Renin Angiotensin System (Devaux et al., 2020), the main role of ACE2 in the GIT is to ensure nutrients absorption. In the GIT, ACE2 functions as a chaperone for the expression of the sodium-dependent neutral amino acid transporter B^0^AT1 (B^0^AT1 binds to the ferredoxin-like domain of ACE2) and amino acid (proline) SIT1 transporters (Camargo et al., 2009; Vuille-Dit-Bille et al., 2015). ACE2, B^0^AT1, and aminopeptidase N form a complex in the brush border membrane of the intestine (Fairweather et al., 2012). It was recently shown that ACE2-B^0^AT1 heterodimers are assembled through the collectrin-like domain of ACE2 (Yan et al., 2020). Although ACE2 is considered to be highly expressed on colonocytes, this viral receptor is poorly expressed on enteroendocrine cells and Paneth cells, and is almost undetectable in goblet cells and tuft cells (Wang et al., 2020). It was previously established that following ACE2 receptor binding, the SARS-CoV-2 spike (S) glycoprotein is processed by a type II transmembrane serine protease, TMPRSS2, prior to membrane fusion. Although both ACE2 and TMPRSS2 are highly expressed in the GIT, the co-expression of these molecules has not been shown on enterocyte, with TMPRSS2 being expressed on ACE2^neg^ intestinal epithelial cells and not mature enterocytes. It should be emphasized that members of the same family, such as TMPRSS4, highly expressed in ACE2^+^ mature enterocytes are probably involved in SARS-CoV-2 S glycoprotein processing (Zang et al., 2020). The enhanced spread of SARS-CoV-2 compared to SARS-CoV-1 has been correlated with the gain of a polybasic furin type cleavage site at the S1/S2 junction in the SARS-CoV-2 S glycoprotein opening the possibility to this glycoprotein to interact with neuropilin1 (NRP-1), a protein known to bind furin-cleaved substrates (Daly et al., 2020; Cantuti-Castelvetri et al., 2020), through interaction with a C-end rule (CendR) terminal motif RRAR_OH_ (Teesalu et al., 2009; Haspel et al., 2011). Both NRP-1 and NRP-2 are expressed in the GIT (Cohen, 2001; Hansel et al., 2004; Yu et al., 2011).

There is evidence indicating that Caco-2 cells, a cell line derived from a human colorectal adenocarcinoma, can serve as model for GIT cells SARS-CoV-2 replication. The replication of SARS-CoV-2 in Caco-2 cells was reported to be comparable to that found in Calu-3 (pulmonary) cells (Chu et al., 2020). It was also reported that Caco-2 cells exposed to vesicular stomatitis virus (VSV) particles pseudotyped with chimeric spike from SARS-CoV-2 that carry receptor binding domain (RBD) from different betacoronaviruses, become infected with VSV particles expressing the RBD from SARS-CoV-2 (Letko et al., 2020). In this study, most other RBDs were incompatible with infection, indicating a requirement for SARS-CoV-2 spike RBD-ACE2 receptor interaction. The requirement for SARS-CoV-2 spike glycoprotein priming by TMPRSS2 during Caco-2 cell infection has been demonstrated using drug-inactivation of TMPRSS2 that partially blocked viral entry (Hoffmann et al., 2020). Caco-2 cells infected with SARS-CoV-2 produce filopodia protrusions containing viral particles (Bouhaddou et al., 2020). However, previous experiments performed in our laboratory indicated that SARS-CoV-2 does not induce a cytopathic effect in Caco-2 cells at least over seven days of cell culture (Wurtz et al., 2021). In the present study we investigate the E-cad expression in Caco-2 cells after exposure to SARS-CoV-2, as well as the effect of infection on tight-, adherens-, and gap-junctions, and the intestinal tissues damages induced by SARS-CoV-2 infection of human ACE2 transgenic mice.

## Materials and Methods

### Cells Culture

The Caco-2 cell line (ATCC^®^ HTB-37 ™) isolated from a human colorectal adenocarcinoma was cultured in a Dulbecco’s Modified Medium F-12 Nutrient Mixture (DMEM F-12) supplemented with 10% Fetal Bovine Serum (FBS; Invitrogen, USA). Caco-2 cells exhibit spontaneous epithelial differentiation *in vitro*. Another human colorectal adenocarcinoma, the HT29 cell line (ATCC^®^ HTB-38 ™), cultured in DMEM F-12 supplemented with 10% FBS and 1% L-glutamine (L-Gln; Invitrogen) was used as control in some experiments.

The Simian Vero-E6 renal epithelial cell line (ATCC^®^ CRL-1586 ™), isolated from *Chlorocebus sabaeus* (African green monkey) was cultured in minimum essential medium (MEM. Gibco; Invitrogen) containing 4% FBS and 1% L-glutamine (L-Gln; Invitrogen). The HCT-8 (ATCC^®^ CCL-244^™^), a human tumor epithelial cell line isolated from an ileal colorectal adenocarcinoma, was cultured in Roswell Park Memorial Institute 1640 medium (RPMI) (Gibco, Thermo Fischer) supplemented with 10% FBS.

All cells were cultured in a 175-cm^2^ flasks at 37°C in a 5% CO_2_ atmosphere. Every two days the medium was replenished, and confluent cultures were sub-cultured after harvesting of adherent cells by trypsination (0,05% Trypsin-EDTA, Invitrogen, USA).

### Virus Production

The SARS-CoV-2 (strain IHUMI-3, lineage B) was previously isolated from the liquid collected from a nasopharyngeal swab. The SARS-CoV-2 isolate was cultured in Vero-E6 cells grown in MEM supplemented with 4% FBS and 1% L-GLn.

The HCoV-OC43 (ATCC^®^ VR-1558), another human coronavirus responsible for the common winter cold (Choi et al., 2021) and able to infect Caco-2 cells (Collins, 1990), was used as a control in this study. This virus was cultured in HCT-8 cells grown in RPMI1640 supplemented with 4% FBS, as described (Owczarek et al., 2018).

The cultures were incubated at 37°C in a 5% CO_2_ atmosphere. After three passages with an almost complete cytopathic effect, the supernatant of each viral culture was collected, centrifuged at 3000 × g for 10 minutes at 4°C, then filtered through a 0.22 µm membrane. The filtrate was made up of 10% FBS and 1% of 2- [4- (2-96 hydroxyethyl) piperazin-1-yl] ethanesulfonic acid (HEPES), and stored at -80°C, to constitute the SARS-CoV-2 and HCoV-OC43 viral stocks.

For mouse infection, Vero E6 cells were cultured at 37°C in DMEM supplemented with 10% FBS, 10 mM HEPES (pH 7.3), 1 mM sodium pyruvate, 1% L-glutamine (L-Gln; Invitrogen). The strain BetaCoV/France/IDF0372/2020 was supplied by the National Reference Centre for Respiratory Viruses hosted by Institut Pasteur (Paris, France). The human sample from which strain BetaCoV/France/IDF0372/2020 was isolated, has been provided from the Bichat Hospital, Paris, France. Infectious stocks were grown by inoculating Vero E6 cells and collecting supernatant upon observation of cytopathic effect; debris were removed by centrifugation and passage through a 0.22-μm filter. Supernatants were stored at -80°C.

### SARS-CoV-2 Replication Kinetics in Caco-2 Cells

The kinetics of viral replication in Caco-2 cells was performed over 72h of infection. For this purpose, cells were distributed in 24-well flat-bottomed plates (Thermo Fisher Scientific) at a concentration of 5×10^5^ cells/mL in DMEM/F-12 supplemented with 10% FBS and 1% L-Glutamine and incubated overnight at 37°C under 5% CO_2_ atmosphere. Infection occurred with an inoculum of 200 µL of SARS-CoV-2 or HCoV-OC43 viruses (MOI of 0.05). After one hour of adsorption at 37°C, the inoculum was removed, and the cells were washed twice with culture medium. Cells were resuspended in 500 µl of DMEM/F-12 medium. 200 µl aliquots were collected 4, 8, 16, 24, 48, and 72h after T0. qRT-PCR performed in triplicates on supernatants assessed the viral release rate. RNA was extracted from 100 µL of cell culture supernatants using the QIAamp 96 Virus QIAcube HT kit (Qiagen). To detect SARS-CoV-2 RNA, real-time RT-PCRs were carried out using N gene primers (Fwd: 5’-GACCCCAAAATCAGCGAAAT-3’, Rev: 5’-TCTGGTTACTGCCAGTTGAATCTG-3’; probes 5’ FAM-ACCCCGCATTACGTTTGGTGGACC-QSY 3’) and the Superscript III Platinium One-step Quantitative RT-qPCR systems with ROX kit (Invitrogen), with a final concentration of 400 nM of primers, 200 nM of probe, in a final volume of 25μl with 5 μl of RNA. The amplification cycles were carried out on a LightCycler 480 (Roche Diagnostics). The ΔCT calculations were performed considering the CT heats of T=0 subtracted from the other times (4, 8, 16, 24, 48, and 72h). The methods of RNA extraction from supernatants of cells inoculated with HCoV-OC43, as well as the qRT-PCR, were the same as described above, replacing the primers for the primers corresponding to this HCV-OC43 (Fwd: 5’-ATGTTAGGCCGATAATTGAGGACTAT-3’; Rev: 5’-AATGTAAAGATGGCCGCGTATT-3’ and probe: 5’ FAM-CATACTCTGACGGTCACAAT-TAMRA 3’).

The viral release was also evaluated by tissue culture infectious dose 50 (TCID_50_). For this purpose, 96-well plates containing 1×10^5^ Vero E6 cell/well were prepared one day in advance and kept overnight in a 5% CO_2_ atmosphere at 37°C. Culture supernatants from Caco-2 cells collected at 4h, 8h, 16h, 24h, 48h, and 72h times were thawed and serially diluted at base ten and inoculated in Vero E6 cells (8 replicates per dilution to a 10^-10^ dilution). The plate were kept at 37°C in a 5% CO_2_ atmosphere for 7 days. Seven days post infection the presence of cytopathic effects was evaluated. The TCID_50_ was calculated according to the Spearman and Kärber algorithm (Ramakrishnan, 2016).

### RNA Extraction and Quantitative-Reverse Transcription Polymerase Chain Reaction (qRT-PCR)

The Caco-2 cell line (2 x 10^5^ cells/well) was cultured in flat-bottom 24-well plates for 12 hours followed by infection with SARS-CoV-2 or HCoV-OC43 at a MOI of 0.5. Twenty-four hours post-infection, RNAs were extracted from cells using a RNeasy Mini Kit (QIAGEN SA) with a DNase I step to eliminate DNA contaminants, according to the manufacturer instructions. The quantity and quality of the RNA was evaluated using a Nanodrop 1000 spectrophotometer (Thermo Science). The first -strand cDNA was obtained using oligo(dT) primers and Moloney murine leukaemia virus-reverse transcriptase (MMLV-RT kit; Life Technologies), using 100 ng of purified RNA. The qPCR experiments were performed using specific oligonucleotide primers and hot-start polymerase (SYBR Green Fast Master Mix; Roche Diagnostics). The amplification cycles were performed using a C1000 Touch Thermal cycler (Biorad). Specific primers used in this study are listed in the **Table 1** and the results of qRT-PCR were normalized using the housekeeping gene β-Actin (ACTB) (Fwd: 5’ CAT GCC ATC CTG CGT CTG GA 3’; Rev: 5’ CCG TGG CCA TCT CTT GCT CG 3’), and expressed as relative expression (2-^ΔCT^), where ΔCt = Ct (Target gene) – Ct(Actin). A similar experimental procedure was chosen to analyze mRNA expression in HT29 cells.

**Table 1:** Primers sequences used for the RT-qPCR.

Genes	Symbols	Primers sequence	
		Sense	Antisense
E-cadherin	CDH1	5’-GAAGGTGACAGAGCCTCTGGA T-3’	5’GATCGGTTACCGTGATCAAAA T-3’
Angiotensin Conversion Enzyme 2	ACE2	5’-CAGGGAACAGGTAGAGGACAT T-3’	5’CAGAGGGTGAACATACAGTTGG-3’
Transmembrane Protease Serine 2	TMPRSS2	5’-AAGTTCATGGGCAGCAAGTG-3’	5’-ACGCCATCACACCAGTTAGA-3’
Transmembrane Protease Serine 4	TMPRSS4	5’-CAAAGTAGAGGCAGGGGAAAA -3’	5’-CGGAAAAAGTTAGGACACAG GA-3’
Neuropilin 1	NRP-1	5’-GCTGGGAAGTGTGTTGATGAC-3’	5’ACAAAGGGGAGAGGAGAGAG AG-3’
Sodium-dependent neutral amino acid transporter	B^0^AT1	5’-GGTGTGTGCCAGTATGATGTTC -3’	5’AAGAGCAGGAAAAGATGAGG TG-3’
α-Disintegrin and metalloproteinase domain-containing protein 10	ADAM-10	5’-AACCTACGAATGAAGAGGGAC A-3’	5’-TGACAGAGTGAAATGGCAGA GT-3’
α-Disintegrin and metalloproteinase domain-containing protein 17	ADAM-17	5’-GCAGGACTTCTTCACTGGACAC -3’	5’-TCTACTAACCCTTTTGGGAGC A-3’
Angiotensin II Receptors 1	AT1R	5’-TGTGGACTGAACCGACTTTTCT -3’	5’-GGAACTCTCATCTCCTGTTGC T-3’
Proto-oncogene Mas	MAS1	5’-GGAGAAAGAGACACCGCATAA C-3’	5’-GTGAAGAGACAGAGAACGA GCA-3’
Proto-oncogene Mas Like	MAS1L	5’-CTGCTCCTGACTGTGATGTTGT-3’	5’-GTTTGGGTTCTGTGCCTCCT-3’
Housekeeping gene β-Actin	ACTB	5’-CATGCCATCCTGCGTCTGGA-3’	5’-CCGTGGCCATCTCTTGCTCG-3’
Platelet endothelial cell adhesion molecule	PECAM-1/CD31	5’-CAAAGACAACCCCACTGAAGAC-3’	5’-TCCAGACTCCACCACCTTACTT-3’
Junctional adhesion molecule A/Platelet adhesion molecule 1	F11R/JAM-A	5’-GGATTTCTCAGGTCATTTGGAG-3’	5’-TAGACTGGTGGATGGTGGTAGA-3’
Connexin-43	Cx43	5’-GGTTCAAGCCTACTCAACTGCT-3’	5’-GTTTCTCTTCCTTTCGCATCAC-3’
Occludin	OCLN	5’-CTTCCATCCTGTGTTGACTTTG-3’	5’-CACTTTTCTGCCCTGATTCTTC-3’
Zonulin	HP2	5’-ATGTGAAGCAGTATGTGGGAAG-3’	5’-AGAGATTTTTAGCCGTGGTCAG-3’
Glyceradehyde-3-Phosphate dehydrogenase	GAPDH	5’-ACACCCACTCCTCCACCTTT-3’	5’-CTCTTCCTCTTGTGCTCTTGCT-3’
Beta-2 microglobulin	B2M	5’-GGTTTCATCCATCCGACATT-3’	5’-GGCAGGCATACTCATCTTTTTC-3’

### Soluble E-Cadherin Quantification

Caco-2 cells (2 x 10^5^ cells/well) were cultured in flat-bottom 12 well plates for 12 hours and were then infected with SARS-CoV-2 or HCoV-OC43 at a MOI (Multiplicity of Infection) of 0.5 for four hours, 24 hours, and 48 hours. For each kinetics, the culture supernatants were collected, centrifuged at 1000g for 10 minutes and stored at -20°C until use. The quantities of sE-cad in the supernatants were determined using a specific immunoassay (sE-cad kit Abcam, Cambridge, UK) according to the manufacturer’s instruction. The minimal detectable concentration of human sE-cad is 156 pg/mL. The quantification of the soluble compounds in the cell culture supernatant was calculated by comparison to standard curves.

### Detection of Protein Expression by Western Blot Analysis

For Western blot assays, cells were immediately washed with ice cold phosphate buffered saline (PBS), and lysed on the plate in a 1X RIPA buffer (100 mM Tris-HCl pH7.5; 750 mM NaCl; 5mM EDTA; 5% Igepal, 0.5% sodium dodecyl sulphate (SDS); 2.5% Na Deoxycholate) supplemented with protease inhibitor and phosphatase cocktail inhibitor (Roche, Germany). Fifty µg of protein was loaded onto a 10% SDS polyacrylamide gels. After the transfer, blockage with a saturation solution (5% Free Fat Milk -PBS-0.3% Tween 20) overnight at 4°C, the blots were incubated with a monoclonal antibody (mAb) directed against human E-cadherin (HECD-131700, Invitrogen, France) for two hours at room temperature. The expression of Glyceradehyde-3-Phosphate dehydrogenase (GADPH) was measured using a mouse anti-human GADPH mAb (1:5,000, Abnova, Taiwan), followed by incubation with a sheep anti-mouse horseradish peroxidase-conjugated antibody (1:10,000 dilution with a blocking solution) (Life Technologies, France) as the loading control. In some experiments the cells were labeled with anti-Occludin mouse mAb (Ref. 331500, Life Technologies SA), anti-connexin 43 mouse mAb (Ref 138300, Life Tech.), anti-JAM-A mouse mAb HU-CD321 (Ref. 14-3219-82, Life Tech.), and/or anti-zonulin mAb (Ref. UM500010, Life Tech.) The proteins were revealed using a ECL Western Blotting Substrate (Promega, USA) and images were digitized using a Fusion FX (Vilber Lourmat, France). The public domain program Image J was used to quantify the Western blot bands intensity.

### Automated Western Immunoblotting for SARS-CoV-2 Nucleocapsid Protein Detection

The Jess™ Simple Western system (ProteinSimple, San Jose CA, USA) is an automated capillary-based size separation and nano-immunoassay system. The antibody-detection of SARS-CoV-2 nucleocapsid protein was performed according to the manufacturer’s standard method for 12-230-kDa Jess separation module (SM-W004), as previously described (Edouard et al., 2021). Briefly, viral proteins were mixed with 0.1X Sample buffer and Fluorescent 5X Master mix (ProteinSimple) to achieve a final concentration of 0.25 μg/μL in the presence of fluorescent molecular weight markers and 400 mM dithiothreitol. After denaturation (95°C for 5 min), the viral proteins were separated in capillaries matrix at 375 volts. A ProteinSimple photoactivated capture chemistry was used to immobilize the proteins on the capillaries and then exposed to antibodies. The chemiluminescent revelation was established with peroxyde/luminol-S (ProteinSimple). Digital image of chemiluminescence of the capillary was captured with Compass Simple Western software (version 4.1.0, Protein Simple) that automatically calculated chemiluminescence intensity, area, and signal/noise ratio.

### Confocal Microscopy Analysis

Caco-2 cells were cultured on sterile coverslips in 24-well plates at an initial concentration of 2×10^5^ cells/well before being infected with SARS-CoV-2 or HCoV-OC43 at a MOI of 0.5 for 24 hours. After fixation with paraformaldehyde (3%), the cells were permeabilized with 0.1% Triton X-100 for three minutes and saturated with 3% BSA- 0,1% Tween 20-PBS for 30 minutes at room temperature. For the primary labelling, cells were incubated for one hour at room temperature with a mix (1:1000 dilution with 3% BSA- PBS-0.3% Tween 20) of mouse monoclonal anti-E-cadherin (4A2C7, Life Technologies, France) directed against the cytoplasmic domain of E-cad and a goat polyclonal anti-ACE2 (MAB933, R&D Systems, Minneapolis, USA). The 4’,6’-diamino-2-fenil-indol (DAPI) (1:2500, Life Technologies) and the Phalloidin (Alexa-488) (1:500, OZYME) were used to stain the nucleus and the filamentous actin, respectively. After washing, cells were incubated for 30 minutes at room temperature with a mix (1:1000) of goat anti-rabbit IgG (H+L) secondary antibody (Alexa Fluor 647) (Life Technologies, France) and donkey anti-goat IgG (H+L) secondary antibody (Alexa Fluor 555) (Life Technologies, France). In some experiments the cells were labeled with anti-occludin mouse mAb (Ref. 331500, Life Technologies SA).

### Electron Microscopy Analysis

Virus-free cells and SARS-CoV-2 infected Caco-2 cells at 48 hours post-infection were prepared as previously described (Le Bideau et al., 2021). Briefly, cells were fixed with glutaraldehyde (2.5%) in a 0.1 M sodium cacodylate buffer. Resin embedding was microwave-assisted with a PELCO BiowavePro+. Samples were washed with a mixture of 0.2 M saccharose/0.1 M sodium cacodylate and post-fixed with 1% OsO4 diluted in 0.2 M potassium hexa-cyanoferrate (III)/0.1 M sodium cacodylate buffer. After washes with distilled water, samples were gradually dehydrated by successive baths containing 30% to 100% ethanol. Substitution with Epon resin was achieved by incubations with 25% to 100% Epon resin, and samples were placed in a polymerization chamber. Resin microwave-curing was performed for a total of two hours. After curing, the resin blocks were manually trimmed with a razor blade and dish bottoms were detached from cell monolayers by cold shock *via* immersion in liquid nitrogen for 20 seconds. Resin blocks were placed in a UC7 ultramicrotome (Leica), trimmed to pyramids, and ultrathin 100 nm sections were cut and placed on HR25 300 Mesh Copper/Rhodium grids (TAAB). Sections were contrasted with uranyl acetate and lead citrate. Grids were attached with double-side tape to a glass slide and platinum-coated at 10 mA for 20 seconds with a MC1000 sputter coater (Hitachi High-Technologies, Japan). Electron micrographs were obtained on a SU5000 SEM (Hitachi High-Technologies, Japan) operated in high-vacuum at 7 kV accelerating voltage and observation mode (spot size 30) with BSE detector.

### Animals

Animal housing and experimental procedures have been conducted according to the French and European Regulations (Parlement Européen et du Conseil du 22 Septembre 2010. Décret n°2013-118 du 1er Février 2013 relatif à la protection des animaux utilisés à des fins scientifiques) and the National Research Council (U.S.), Institute for Laboratory Animal Research (U.S.), and National Academies Press (U.S.), Eds., Guide for the care of laboratory animals, 8th ed. Washington, D.C: National Academies Press, 2011. The CIPHE BSL3 facility operates under Agreement N° B 13 014 07 delivered by the French authorities. All animal procedures (including surgery, anesthesia and euthanasia as applicable) used in the current study have been submitted to the Institutional Animal Care and Use Committee of CIPHE approved by French authorities (CETEA DSV -APAFIS#26484-2020062213431976 v6). All the CIPHE BSL3 facility operations are oversee by a Biosecurity/Biosafety Officer and accredited by Agence Nationale de Sécurité du Médicament (ANSM). K18-hACE C57BL/6J mice (strain: 2B6.Cg-Tg (K18-ACE2)2Prlmn/J) were obtained from The Jackson Laboratory. Animals were housed in groups and fed standard chow diets.

### Infection of K18-hACE2 Transgenic Mice

8-12 weeks old K18-hACE2 transgenic mice (human ACE2+), kindly provided by The Jackson laboratory, were infected with 2.5x10^4^ PFU of Wuhan/D614 SARS-CoV-2 (BetaCoV/France/IDF0372/2020) *via* intranasal administration. Mice were monitored daily for morbidity (body weight) and mortality (survival). During the mice monitoring period, mice were scored for clinical symptoms (weight loss, eye closure, appearance of fur, posture, and respiration). Mice showing clinical scoring defined as reaching experimental end-point were humanely euthanized.

### Histological Analysis

Each mouse intestine removed was fixed with buffered formalin at 4% and embedded in paraffin. Serial sections (3 μm) of these specimens were obtained for hematoxylin-phloxin-saffron staining. Briefly, the presence of intestinal lesions was determined after a complete optical examination of at least three sections of intestine tissues from each K18-hACE2 transgenic mice (n=3) using the image analyzer NDP.view2 Viewing Software UT12 388-01 (Hamamatsu, Japan).

### Statistical Analysis and Correlation Analysis

Analysis of variance (ANOVA) were performed using the GraphPad-Prism software (version 6.01). Data were analyzed using a one or a two-way ANOVA with the Holm-Sidak multiple *post-hoc* test for group comparison. A p-value <0.05 was considered statistically significant. The results are presented as the mean with standard error of the mean (SEM). The data were also submitted to multivariate principal component analysis (PCA) and hierarchical clustering heatmap analysis using the ClustVis software (https://biit.cs.ut.ee/clustvis/).

## Results

### Caco-2 Cells, a Suitable Cellular Model for Studying the *CDH1*/E-Cadherin Gene and E-Cad Proteins Expression

Our main purpose was to compare the expression of both the *CDH1* gene (encoding E-cadherin mRNA) and E-cad proteins in virus-free Caco-2 cells and SARS-CoV-2 infected Caco-2 cells. This study was carried out in order to question the ability of the virus to modulate the expression of this cell adhesion molecule known to play a central role in the integrity of intestinal cell-to-cell adherens junctions. Caco-2 cells were previously reported to be a model of human intestinal cells susceptible to SARS-CoV-2 infection and able to support the *de novo* production of viral particles. However the expression of cellular genes coding for the viral receptor/co-receptor, had not thus far been quantified in these cells. It includes: i) ACE2 allowing the binding of the viral spike (S) and entry of the virus into the cell; ii) the cellular serine proteases TMPRSS2 and TMPRSS4 reported to be required for the S glycoprotein priming; and, iii) Neuropilin-1 (NRP-1), also described as able to potentiate SARS-CoV-2 entry. As a result, before investigating the transcriptomic response of Caco-2 cells to SARS-CoV-2 infection, the first part of this study thus consisted of estimating the expression of these genes in Caco-2 cells cultured in virus-free medium. Our objective was also to confirm the physiological relevance of the Caco-2 cells cellular model by analyzing the basal expression of the *CDH1*/E-cad gene to ensure that it was sufficiently expressed in those cells to be able to quantify a possible down modulation of this gene after infection. The basal expression of other genes that could possibly be involved in cell-surface receptor mediated modulation of *CDH1*/E-cad gene was also quantified.

**Figure 1:**
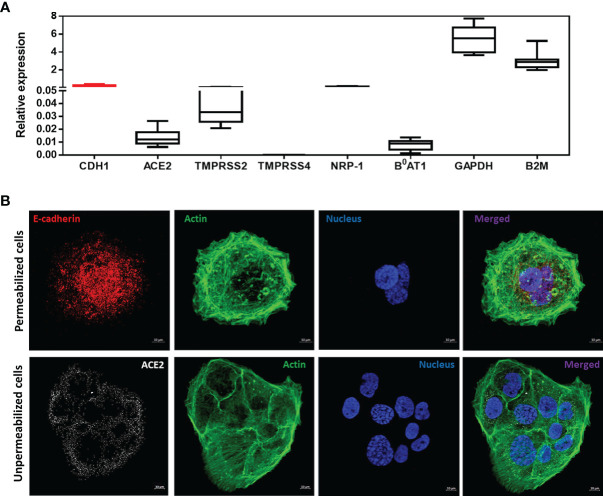
**(A)** Expression of CDH1/E-Cad, ACE2, TMPRSS2, TMPRSS4, NRP-1, B^0^AT1, GAPDH and B2M mRNA in virus-free Caco-2 cells. The results (n=8) are expressed as RE where RE = 2^(−ΔCT)^. **(B)** Confocal microscope analysis of E-cad, ACE2 and actin expression on Caco-2 cells. The experiment was performed using cells permeabilized with 0.1% Triton X-100 (upper panel) and unpermeabilized cells (lower panel).

As shown in **Figure 1A**, the qRT-PCR analysis indicated a very high expression of *CDH1*/E-cad mRNAs in virus-free Caco-2 cells. Regarding the genes coding for the ACE2 receptor and its intestinal ligand B^0^AT1, the mRNAs encoding these proteins are detectable, although their expression is relatively low. As for the expression of mRNAs encoding the TMPRSS2 and TMPRSS4 proteases, we found that the gene encoding the TMPRSS2 protein is well expressed on Caco-2 cells. In contrast, we noted an absence of detectable expression of the TMPRSS4 mRNA. Finally, there is a high expression of the NRP-1 receptor which possibly acts as a co-receptor for SARS-CoV-2 at the intestinal level.

These results were then confirmed by using confocal imaging of Caco-2 cells labeled with anti-E-cad and anti-ACE2 antibodies and phalloidine to stain the nucleus and the filamentous actin as control (**Figure 1B**). Under these experimental conditions we also observed a high expression of E-cad protein in Caco-2 cells, and the intensity of fluorescence observed with the anti-E-cad labeling was above that of anti-ACE2 labeling.

ACE2 is a peptidase known to control the hydrolysis of angiotensin II (Ang II) into Ang-(1-7), and Ang-(1-7) inhibits the PAK1/NF-KB/Snail 1 signaling pathway *via* activation of the MAS receptor leading to E-cad expression (Yu et al., 2016). In cell types regulated by Ang-(1-7), when the Ang-(1-7) concentration is insufficient the signaling pathway is activated and results in the inhibition of E-cad expression. To ensure that in Caco-2 cells such complex regulation does not interfere with E-Cad expression, we also investigated the expression of mRNAs coding for the Ang II and Ang-(1-7) receptors AT1R, MAS-1, and MA1-L, respectively and found that they are almost undetectable in Caco-2 cells, suggesting that they are unlikely to interfere with Caco-2 cell signaling (**Supplementary Figure 1**).

Taken as a whole, these data indicate that Caco-2 is a suitable cellular model for studying the *CDH1*/E-cadherin gene and E-cad proteins expression in intestinal epithelial cells

### SARS-CoV-2 Replicates in Caco-2 Cells and Modulates ACE2 Transcription and Protein Expression

**Figure 2:**
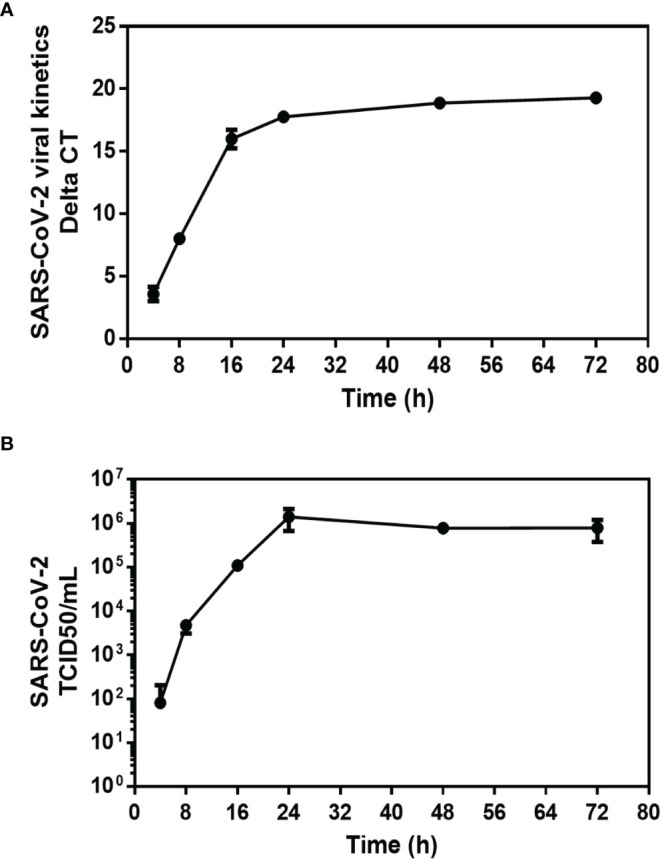
Kinetics of SARS-CoV-2 infection in Caco-2 cells. After viral inoculation in Caco-2 cells, the viral release was monitored over 72 hours (T=0, 4h, 8h, 16h, 24h, 48h, and 72h) from the cell’s supernatant. **(A)** Quantification of the viral release from Caco-2 cell by qRT-PCR: ΔCT represents the CT value obtained subtracted from the CT value at time T=0 (CT - CT_0_), where T=0 is the moment immediately after removing the inoculum used in the adsorption step. Each value is the mean of triplicates **(B)** Quantification of SARS-CoV-2 infectious particles released in the supernatant of Caco-2. The TCID_50_ was performed by inoculating supernatants collected from SARS-CoV-2 infected Caco-2 cells into a culture of Vero E6 cells. The cytopathic effect (CPE) was evaluated on Vero E6 cells at 7 days after exposure to Caco-2 culture supernatants. Each value is the mean of triplicates. The TCID_50_/mL is calculated according to the Spearman and Kärber algorithm.

**Figure 3:**
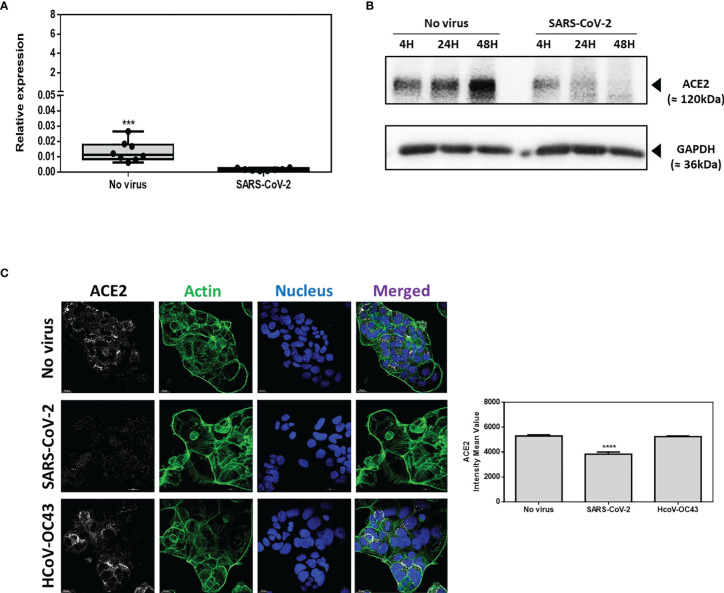
ACE2 expression on Caco-2 cells. **(A)** ACE2 mRNA in virus-free Caco-2 cells or cells exposed for 24h to SARS-CoV-2 or HCoV-OC43 at MOI of 0.5. The results are expressed as RE where RE = 2^(−ΔCT)^. **(B)** Time course (4h, 24h, 48h) Western blot analysis of ACE2 proteins expression in virus-free Caco-2 cells or cells exposed to SARS-CoV-2 at an MOI of 0.5. **(C)** Illustration of single plane confocal microscope analysis of ACE2 expression on Caco-2 virus-free cells or cells exposed for 24h to SARS-CoV-2 or HCoV-OC43 at MOI of 0.5. The analysis was performed on unpermeabilized cells. Actin expression was shown as control as well as the labeling of the nucleus (left panel). Quantitative representation (n=4) of mean fluorescence intensity corresponding to ACE2 protein expression on Caco-2 cells infected or not with SARS-CoV-2 or HCoV-OC43 (right panel). The symbol ****means a p-value < 0.0001; The symbol *** means a p-value <0.001.

In order to confirm that the Caco-2 cells model is suitable to analyze the transcriptional response of genes of interest in intestinal epithelial cells infected with SARS-CoV-2, Caco-2 cell infection by SARS-CoV-2 has been precisely characterized. Cells were cultured either in the absence of virus (control) or in the presence of SARS-CoV-2 at a MOI of 0.5. To confirm the infection of Caco-2 cells with SARS-CoV-2, the kinetics of viral replication in Caco-2 cells exposed to SARS-CoV-2 was quantified using a qRT-PCR amplification of the SARS-CoV-2 N gene performed on cell culture supernatants of Caco-2 cells over 72h of infection (**Figure 2A**). The qRT-PCR data were considered positive when the cycle threshold (Ct) was below 30. At T=0 the Ct was 37. After 4h incubation the culture supernatants of Caco-2 cells remained negative (Ct=33.42; ΔCt=3.58) and started to be positive at 8h post-infection (Ct=28.98; ΔCt=8.02). The culture supernatants of Caco-2 cells became clearly positive at 16h post-infection with a Ct=21.02 (ΔCt=15.98) and then reached a plateau at 24h post-infection with ΔCt=17.76, ΔCt=18.86 and ΔCt=19.28 using 24h, 48h and 72h culture supernatants of SARS-CoV-2 exposed Caco-2 cells, respectively. The viral release was also estimated by the observation of the cytopathic effect (CPE) on Vero E6 cells after 7 days incubation with culture supernatants from Caco-2 cells collected at 4h, 8h, 16h, 24h, 36h, 48h and 72h after viral exposure (**Figure 2B**). The CPE was monitored under a photonic microscopy to determine the virus TCID_50_. The culture supernatant from Caco-2 cells collected 4h after viral exposure gave a TCID_50_ of 8.07 x 10^1^/mL while the culture supernatants from Caco-2 cells collected 24h post infection gave a value of 1.39 x 10^6^ TCID_50_/mL. The plateau of CPE on Vero E6 cells was reached using Caco-2 cells culture supernatants collected after 24h of infection. These results were confirmed by the detection of the viral nucleocapsid using the high-speed capillary electrophoresis technologies that revealed the presence of the viral protein synthesis 24h post-infection (data not shown). Moreover, we also compared the viral receptor (ACE2) mRNA expression in virus-free Caco-2 cells and SARS-CoV-2-infected Caco-2 cells by qRT-PCR monitoring. We found a significantly (p<0.001) lower expression of the mRNA encoding ACE2 in cells infected with SARS-CoV-2 compared with SARS-CoV-2-free Caco-2 cells (**Figures 3A** **)**. Regarding the expression of ACE2 protein it also undergoes a decrease in Caco-2 cells after exposure to SARS-CoV-2 (**Figures 3B**). This time course analysis demonstrated that the ACE2 production is markedly reduced 24 h after infection with SARS-CoV-2 and becomes almost undetectable at 48 h post-infection. This was further confirmed by a confocal microscopy analysis which highlights a significant (p-value <0.0001) down-regulation of ACE2 when Caco-2 cells were exposed to SAR-CoV-2 (**Figures 3C**). In contrast, no modulation of ACE2 expression was found when Caco-2 cells were exposed to the human betacoronavirus HCoV-OC43 that uses a different receptor (the ubiquitous HLA class I receptor, present on all human cells including intestinal cells) to enter cells. This is consistent with previous reports indicating that SARS-CoV-2 induces the modulation of its cell surface receptor ACE2 during the infection of cells.

**Figure 4:**
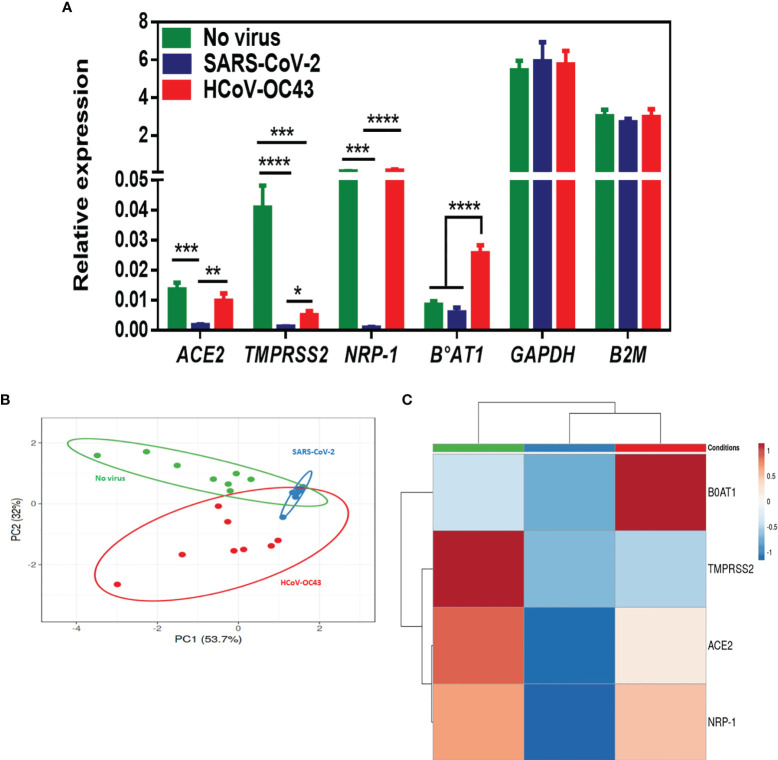
**(A)** mRNA expression of ACE2, TMPRSS2, NRP-1, B^0^AT1, GAPDH, and B2M in virus-free Caco-2 cells or cells exposed to coronaviruses SARS-CoV-2 or HCoV-OC43 at a MOI of 0.5 for 24 hours. The results (n=8) are expressed as RE where RE = 2^(−ΔCT)^. **(B, C)** PCA and hierarchical clustering heatmap analysis of different molecules expressed on Caco-2 cells in virus-free cells, and cells exposed to coronaviruses SARS-CoV-2 or HCoV-43. The symbol *** means a p-value < 0.001; the symbol **** means a p-value < 0.0001.

To complete the transcriptional analysis of Caco-2 cells after SARS-CoV-2 infection, in addition to ACE2 we also performed a qRT-PCR monitoring of TMPRSS 2 and TMPRSS 4, NRP- 1 and B^0^AT1. As shown in **Figure 4A**, we found that there is a significant lower expression of TMPRSS2 and NRP-1 mRNA in Caco-2 cells infected with SARS-CoV-2 compared to the basal levels of expression of these mRNAs in SARS-CoV-2-free Caco-2 cells. Finally, we also observed a lower expression of mRNAs encoding the protein B^0^AT1. The comparison of pattern between SARS-CoV-2-, and HCoV-OC43-exposed Caco-2 cells indicated a difference of ACE2 and NRP-1 genes regulation by these two viruses, with a lack of ACE2 and NRP-1 mRNAs expression modulation during HCoV-OC43 infection of Caco-2 cells. Surprisingly, we observed an increased expression of mRNAs encoding the protein B^0^AT1 in HCoV-OC43-exposed Caco-2 cells which is not accompanied by the modulation of ACE2 expression. According to the principal component analysis and hierarchical clustering heatmap (**Figures 4B, C**), it appears that the expression of cellular genes in virus-free conditions segregated differently from the conditions with SARS-CoV-2-, and HCoV-OC43-exposed Caco-2 cells.

### Modulation of CDH1/E-Cad Expression by SARS-CoV-2 in Caco-2 Cells

**Figure 5:**
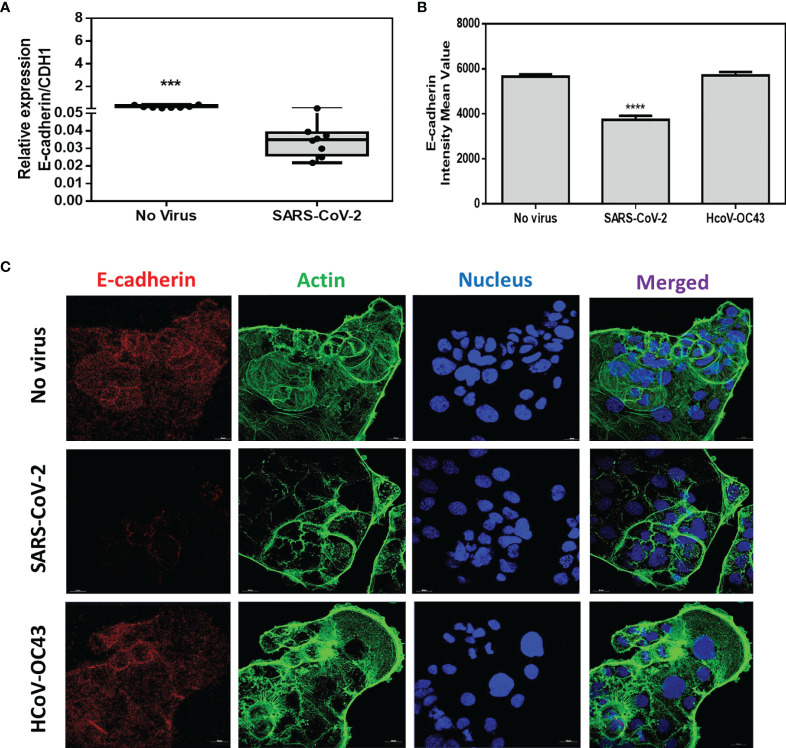
Expression of CDH1/E-cad mRNA and E-cad protein. **(A)** Expression of CDH1/E-cad mRNA in virus-free Caco-2 cells or cells exposed to SARS-CoV-2 at an MOI of 0.5 for 24 hours. The results (n=8) are expressed as RE where RE = 2^(−ΔCT)^.The symbol *** means a p-value < 0.001. **(B)** Quantitative representation of mean fluorescence intensity (n=4) corresponding to E-cad protein expression on Caco-2 cells infected or not with SARS-CoV-2 or HCoV-OC43. The symbol the symbol ****means a p-value < 0.0001. **(C)** Illustration of single plane confocal microscope analysis of E-cad expression on subconfluent virus-free Caco-2 cells or cells exposed to coronaviruses SARS-CoV-2 or HCoV-43 at an MOI of 0.5 for 24 hours. The analysis was performed on cells permeabilized with 0.1% Triton X-100. Actin expression was shown as control as well as the labeling of the nucleus.

Then, we studied the effects of SARS-CoV-2 infection on CDH1/E-cad gene expression. We performed a qRT-PCR monitoring of the CDH1/E-cad mRNA expression in virus-free Caco-2 cells and SARS-CoV-2-infected Caco-2 cells. The qRT-PCR analysis (**Figure 5A**) revealed a significantly (p<0.001) lower expression of the mRNA encoding E-cad in cells infected with SARS-CoV-2 compared with SARS-CoV-2-free Caco-2 cells. To confirm this result, confocal immunofluorescence analysis was performed. As shown in **Figures 5B, C**, a significantly (p<0.0001) lower expression of E-cad was observed in SARS-CoV-2 infected cells when compared to virus-free Caco-2 cells. As an additional control, we performed similar experiments in the presence of HCoV-OC43, which does not depends on ACE2 interaction to infect cells. No decrease in E-cad protein expression was observed in Caco-2 cells exposed to HCoV-OC43.

### Increased Release of Soluble E-Cad in the Culture Supernatant of SARS-CoV-2-Infected Caco-2 Cells

**Figure 6:**
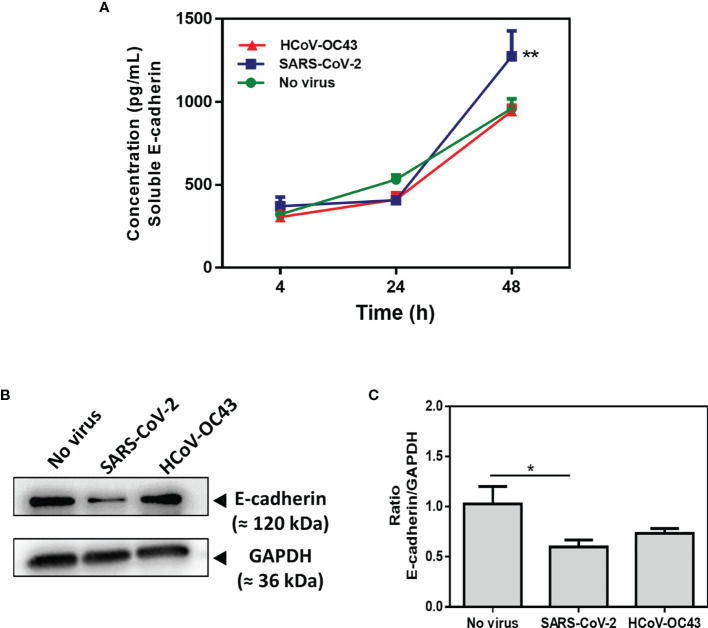
Expression of E-cad and sE-cad. in Caco-2 cells. **(A)** Quantification of sE-cad (n=4) in the cell-culture supernatant of virus-free Caco-2 cells and cells exposed to coronaviruses SARS-CoV-2 or HCoV-OC43 at an MOI of 0.5 for 4 hours, 24 hours and 48 hours respectively, using antigen-specific ELISA. The results are the average of quadruplicates. **(B)** Western blot analysis of E-cad expression in virus-free Caco-2 cells and cells exposed for 48h to SARS-CoV-2 or HCoV-OC43 coronaviruses. GAPDH was used as loading control. **(C)** Quantitative representation of E-cad protein expression by Caco-2 cells infected or not with SARS-CoV-2 or HCoV-OC43 normalized with respect to GAPDH. The Image J program was used to quantify the Western blot bands intensity. The symbol ** means a p-value < 0.01. The symbol * means a p-value < 0.05.

Due to the observed modulation of E-cad expression in cells infected with SARS-CoV-2, we wanted to study the possible over-release of sE-cad in the cell culture supernatant of Caco-2 infected with the virus. In several infection processes initiated in the GIT lumen, a soluble form of E-cad, sE-cad is released from the cell membrane after cleavage of the integral protein by sheddases, thereby disrupting the intercellular junctions required for epithelial cell barrier stability (e.g., this leads to pathogen transmigration). The concentration of sE-cad in cell culture supernatant (which naturally increase with time in cell culture) was measured using an ELISA (**Figure 6A**). We notice a significant (p<0.01) increase in sE-cad release in Caco-2 cells infected with SARS-CoV-2 at 48 h post-infection (mean value: 1274.65pg/mL), compared to the virus-free cell control (mean value: 961.42pg/mL). In contrast, we found no increase in sE-cad release in Caco-2 cells infected by HCoV-OC43 control (mean value: 944.92pg/mL). These results were confirmed by Western Blot on which we observed a significant (p<0.05) decrease in the amounts of the 120 kDa E-cad in the condition of infection with SARS-CoV-2 compared to virus-free cells and Caco-2 cells exposed to HCoV-OC43 (**Figures 6B, C**).

**Figure 7:**
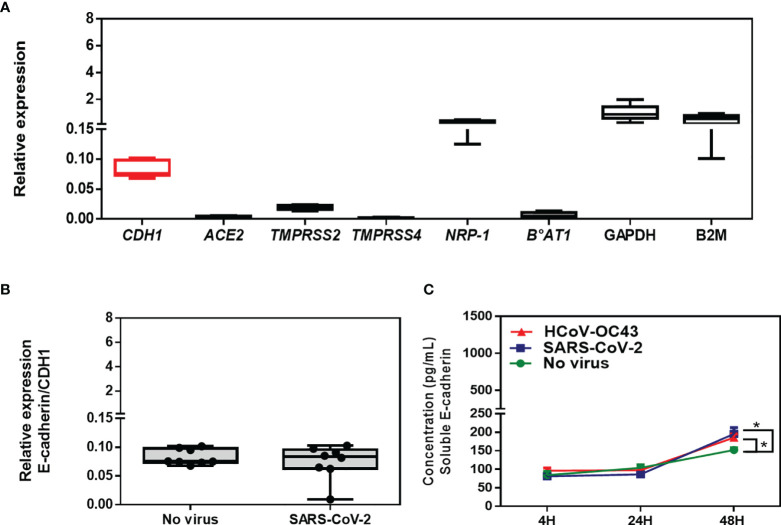
**(A)** Expression of CDH1/E-Cad, ACE2, TMPRSS2, TMPRSS4, NRP-1, B^0^AT1, GAPDH, and B2M mRNA in virus-free HT29 cells. The results (n=8) are expressed as RE where RE = 2^(−ΔCT)^. **(B)** Expression of CDH1/E-cad mRNA in virus-free HT29 cells or cells exposed to SARS-CoV-2 at an MOI of 0.5 for 24 hours. **(C)** Quantification of sE-cad (n=4) in the cell-culture supernatant of virus-free HT29 cells and cells exposed to coronaviruses SARS-CoV-2 or HCoV-43 at an MOI of 0.5 for 4 hours, 24 hours and 48 hours respectively, using antigen-specific ELISA. The symbol * means a p-value < 0.05.

In order to verify whether this effect of SARS-CoV-2 on the expression of E-cad adherens junctions proteins is specific to the Caco-2 cell line or more generally affects the cells of the intestinal epithelium, the experiment was reproduced using the mucin-producing human colorectal adenocarcinoma HT29 cell line. Using qRT-PCR for phenotyping of genes expression in virus-free HT29 cells we found that expression of ACE2 and B^0^AT1 were very low, while TMPRSS2, NRP-1 and *CDH1*/E-cad were highly expressed in these cells (**Figure 7A**). Although the basal level of expression of the *CDH1*/E-cad gene remained quite stable in HT29 cells exposed to SARS-CoV-2 (**Figure 7B**) and viral release was too low to be quantifiable in the culture supernatants of HT29 cells while SARS-CoV-2 proteins can be detected in these cells using high speed capillary electrophoresis (**Supplementary Figure 2**), a significantly (p<0.05) higher release of sE-cad in the supernatant of HT29 cells exposed to SARS-CoV-2 was also found when compared to the uninfected control. In those cells, HCoV-OC43 also induced sE-cad release (**Figure 7C**).

### Probable Involvement of Human Sheddases in SARS-CoV-2-Induced Release of Soluble E-Cad From Caco-2 Cells

**Figure 8:**
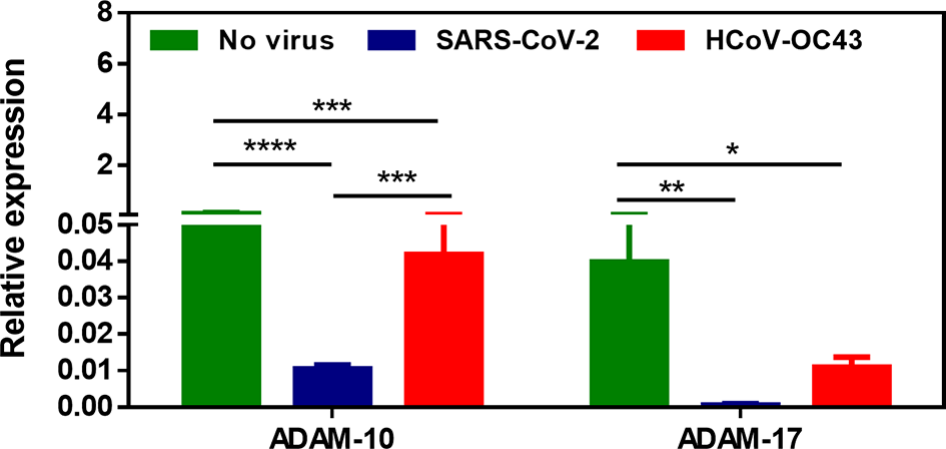
Expression of ADAM-10 and ADAM-17 sheddases mRNA in virus-free Caco-2 cells or cells exposed to SARS-CoV-2 or HCoV-OC43 coronaviruses at an MOI of 0.5 for 24 hours. The results (n=8) are expressed as RE where RE = 2^(−ΔCT).^ The symbol * means a p-value < 0.05; the symbol ** means a p-value < 0.01; the symbol *** means a p-value < 0.001; the symbol **** means a p-value < 0.0001.

Several sheddases have been reported able to lead to sE-cad release from cells during infectious processes resulting in intestinal tissues damages (Devaux et al., 2019). This is why we postulated that cellular sheddases could be induced to cleave E-cad in SARS-CoV-2 infected cells (the best known candidate to achieve cleavage of E-cad in the intestinal tract being ADAM-10, while ADAM-17, another member of the same family of proteases, is known to cleave ACE2), and compared the expression of ADAM-10 and ADAM-17 genes in Caco-2 cells following viral infection with their expression in virus-free cells. This experiment revealed a significantly (p < 0.0001) lower expression of ADAM-10 as well as ADAM-17 (p<0.01) sheddases in Caco-2 cells infected with SARS-CoV-2 (**Figure 8**). Expression of these sheddases was also reduced in HCoV-OC43 infected cells. Similar experiments were performed using other cellular sheddases, including ADAM-8, ADAM-15, MMP-3, MMP-9, MMP-12 and HtrA1. We found that the expression of most of these sheddases is lower in Caco-2 cells infected by SARS-CoV-2, although the expression of ADAM-15 expression was not modulated after SARS-CoV-2 infection (data not shown). The possible association between SARS-CoV-2 infection, sE-cad release, and the expression of sheddases remains to be further investigated in order to identify the sheddase(s) responsible for the cleavage of the membrane E-cad during SARS-CoV-2 infection of intestinal epithelial cells.

### Effect of SARS-CoV-2 Infection on Expression by Caco-2 Cells of Molecules Involved in Tight Junctions and Gap Junctions

E-cad is a major component of the so-called adherens junctions. In order to investigate whether SARS-CoV-2 triggers specific modulation of E-cad or more widely affects molecules required to maintain junctions between neighboring epithelial cells, we investigated the result of SARS-CoV-2 infection of Caco-2 cells on molecules (occludin, zonulin and connexin-43), involved in tight junctions or gap junctions. Intestinal epithelial cells express four types of junctional proteins that bind the cells together (Meenan et al., 1996; Chelakkot et al., 2018) and consist of: i) occluding junctions (zonula occludens or tight junctions), generated by the assembly of multiple integral transmembrane proteins (occludin, claudins, junctional adhesion molecule/JAM, and tricellulin) located near the apical part of the epithelium between neighboring cells (they maintain intestinal barrier function and control cell polarity and the permeability of the paracellular transport pathway) and peripheral membrane adaptor proteins (zonula occludens ZO-1, ZO-2, and ZO-3) acting as bridges to connect integral membrane proteins to the actin cytoskeleton; ii) adhering junctions (zonula adherens) which lies below the tight junction are composed of cadherins (E-cad) from adjacent cells that act to ‘zipper’ up together the gap between two adjacent cells playing a role in the morphogenesis of epithelial cells; iii) desmosomes (macula adherens) that lie on the basal membrane, to help stick the cells to the underlying basal lamina and involve keratin the function of which is to withstand abrasion; and, iv) gap junctions that are communicating junctions (also known as nexus) involving a group of proteins called connexins which form a continuous channel between adjacent cells. In addition, another cell adhesion protein, PECAM, is expressed in the basolateral epithelial membrane and is involved in the migration of leucocytes across the connective tissues.

**Figure 9:**
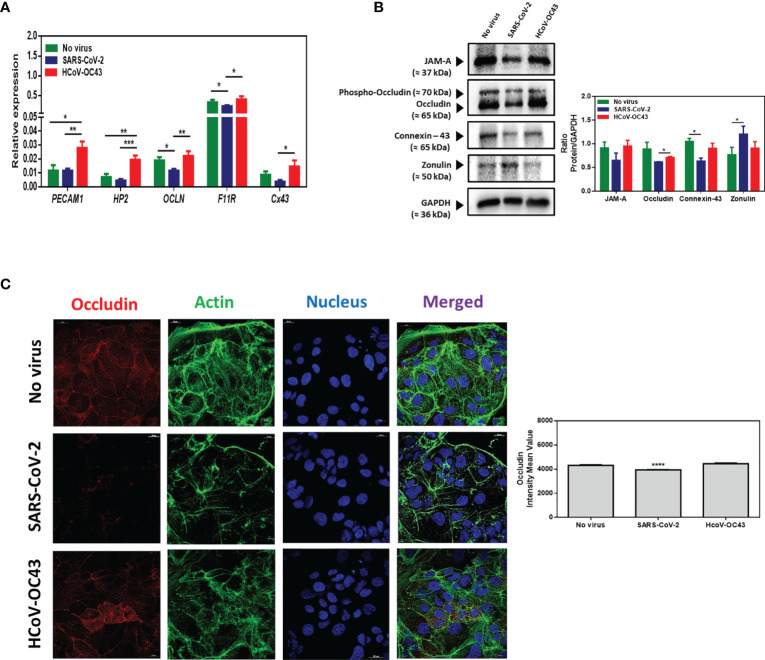
**(A)** mRNA expression of PECAM1, HP2, OCLN, F11R and Cx43, in virus-free Caco-2 cells or cells exposed to coronaviruses SARS-CoV-2 or HCoV-OC43 at a MOI of 0.5 for 24 hours. The results (n=8) are expressed as RE where RE = 2^(−ΔCT)^ **(B)** Western blot analysis (left panel) of JAM-A, occludin, connexin-43, and zonulin expression in virus-free Caco-2 cells and cells exposed for 48h to SARS-CoV-2 or HCoV-43 coronaviruses. GAPDH was used as loading control. The quantitative representation (Image J program) of these proteins expression is also illustrated (right panel). **(C)** Illustration of single plane confocal microscope analysis of occludin expression on subconfluent Caco-2 cells grown in RPMI1640 supplemented with 4% FCS only or exposed to coronaviruses SARS-CoV-2 or HCoV-43 at an MOI of 0.5 for 24 hours (left panel). The analysis was performed on unpermeabilized cells. Actin expression was shown as control as well as the labeling of the nucleus. A quantitative representation of mean fluorescence intensity corresponding to occludin protein expression on Caco-2 cells infected or not with SARS-CoV-2 or HCoV-OC43 is shown (right panel). The symbol * means a p-value < 0.05; ** means a p-value < 0.01; *** means a p-value < 0.001; **** means a p-value < 0.0001.

**Figure 10:**
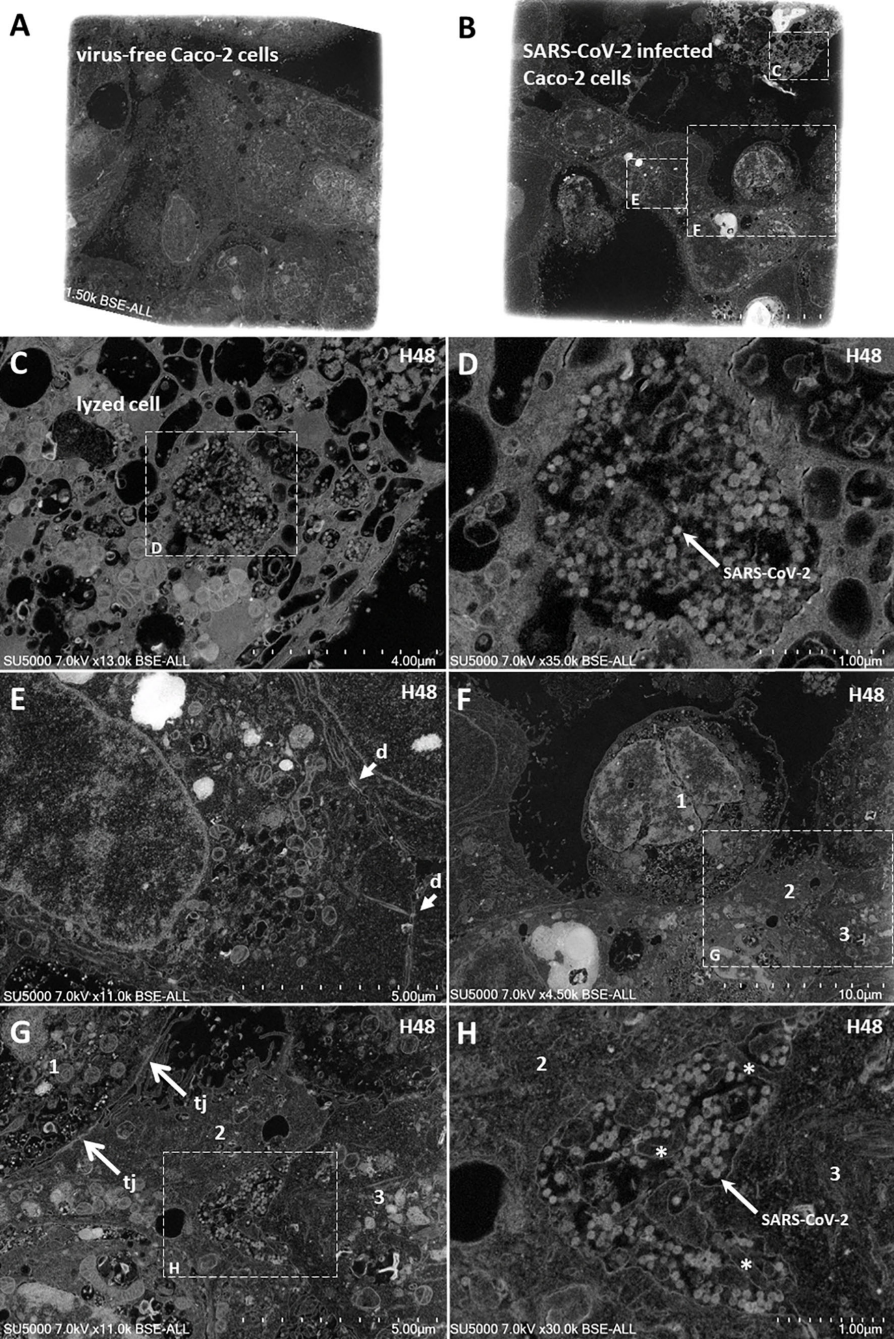
Scanning electron microscopy of SARS-CoV-2 infected Caco-2 cells. **(A, B)** Low-magnification images of virus-free Caco-2 cells monolayer at 0 hours-post-infection (H0; **A**) and SARS-CoV-2 infected Caco-2 cells monolayer at 48 hours-post-infection (H48; **B**). **(C)** Zoom-in boxed region from **(B)** with a lysed cell detached from the cell monolayer. **(D)** Zoom-in boxed region in lysed cell **(C)** with Sars-CoV-2 particles. **(E)** Intact stitched cells from the monolayer with presence of desmosomes (d; arrows) at cell-cell membranous contacts. **(F)** a cell (1) at the center, partially detached from surrounding cells. **(G)** partially detached cell (1) contacting cell (2) *via* tight junctions (tj; arrows). **(H)** Sars-CoV-2 particles located in the monolayer between intact cells (2) and (3), with presence of microvilli (asterisks).

We performed a qRT-PCR for monitoring the expression of *OCLN* gene encoding the occludin mRNA, *F11R* gene encoding the JAM-A mRNA, and the *Cx43* gene encoding the connexin-43 as well as *HP2* encoding zonulin and *PECAM1* encoding PECAM/CD31. We found that *OCLN*, and *F11R* genes encoding tight junctions molecules are highly expressed in Caco-2 cells while the *PECAM1*, *HP2*, and *Cx43* are poorly expressed in this cell line. Then, the mRNA expression of the different cell adhesion molecules was evaluated in the presence or absence of SARS-CoV-2 infection. As shown in **Figure 9A**, the expression of *OCLN* and *F11R* genes expression was found significantly lower in Caco-2 cells infected with SARS-CoV-2 compared to the basal levels of expression of these mRNAs in SARS-CoV-2-free Caco-2 cells. The expression of the Cx-43 gene was apparently also reduced (but it was not statistically significant) while the expression of the other genes tested remained stable. Moreover, we studied the expression of the corresponding proteins by western blot analysis. As shown in **Figures 9B**, the expression of JAM-A and occludin was apparently reduced in SARS-CoV-2 infected cells (but it was not statistically significant) when compared to virus-free Caco-2 cells, indicating that the SARS-CoV-2 infection dysregulates the expression of molecules required for the integrity of tight junctions. This result was further confirmed by confocal microscopy analysis of occludin expression in SARS-CoV-2 infected Caco-2 cells (**Figure 9C**). Under such experimental conditions we found a significant (p<0.0001) down-regulation of occludin. In Caco-2 cells, the expression of connexin-43 (gap junctions) was significantly (p<0.05) down-regulated by the SARS-CoV-2 infection. Interestingly, a significant (p<0.05) increase in zonulin was also observed in SARS-CoV-2 infected Caco-2 cells, suggesting increased GIT permeability during infection. Altogether, these data indicate that SARS-CoV-2 infection not only dysregulates the expression of E-cad required for adherens junctions but also affects several other molecules involved in intercellular adhesion. This is consistent with the observation that 48 hours post-infection with SARS-CoV-2, viral particles can be observed in the intercellular space of adjacent Caco-2 cells by scanning electron microscopy (**Figure 10**). Moreover, lysed cells containing SARS-CoV-2 particles are seen detached from the monolayer. Yet, membranous cell-cell contacts such as tight junctions are still found at contact zones between subsets of detaching cells and cells remaining intact within the monolayer, and desmosomes can be clearly depicted between intact cells in the monolayer.

### SARS-CoV-2 Infection in Human ACE2 Transgenic Mice Leads to Intestinal Tissues Damage

**Figure 11:**
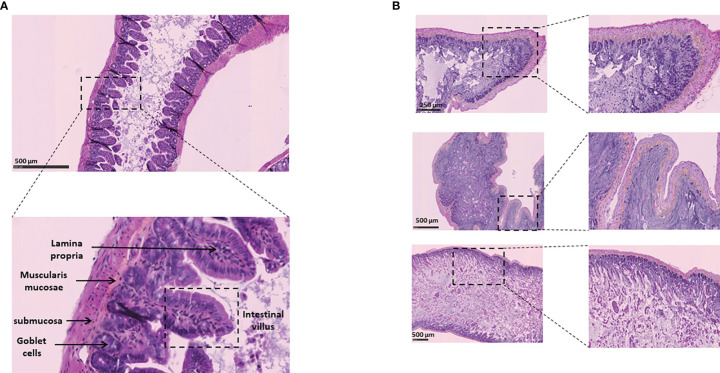
Histological analysis of intestinal tissues from K18-hACE2 transgenic mice (virus free) and K18-hACE2 transgenic mice infected *via* intranasal administration of SARS-CoV-2. **(A)** Normal intestinal wall from control K18-hACE2 transgenic mouse (hematoxylin-phloxin-saffron staining). **(B)** Section of intestine from K18-hACE2 transgenic mouse infected with SARS-CoV-2. The upper, middle and lower panels correspond to samples from 3 different mice. Note the extensive necrosis and inflammation of the lamina propria of intestinal villi with degeneration of intestinal epithelial cells in the intestinal lumen.

Based on our *in vitro* data, we hypothesized that SARS-CoV-2-infected mice may exhibit virus-induced histological lesions. To this end, 8-12 weeks old K18-hACE2 transgenic mice were infected *via* intranasal administration of SARS-CoV-2 and maintained in solitary confinement in a BSL3 animal facilities. Mice with clinical symptoms (weight loss, eye closure, appearance of fur, prostrate posture, and difficulty of breathing) were humanely euthanized and intestine sample were collected. As shown in **Figure 11**, hematoxylin-phloxin-saffron staining to sections of intestine from the virus-free mice showed a normal tissue architecture while sections of intestine from K18-hACE2 transgenic mice infected *via* intranasal administration of SARS-CoV-2 showed a necrotizing enteritis, with sloughing and disruption of the normal architecture, necrosis of the lamina propria of intestinal villi with edema and leukocytic infiltration. These *in vivo* observations are consistent with the dysregulation of intercellular tight junctions, adherens junctions, and gap junctions leading to intestinal permeability.

## Discussion

In this study, we demonstrate that SARS-CoV-2 infection of non/low-mucus producing human colorectal adenocarcinoma Caco-2 cells (Navabi et al., 2013) results in the modulation of the *CDH1* gene encoding the E-cad adhesion molecule as well as several other genes, including the gene encoding the virus receptor, ACE2. This modulation in the expression of *CDH1*/E-cad is accompanied by a lower surface expression of the E-cad protein and by a significant increase in the release of sE-cad into the culture medium. Our data corroborate the observations recently published by Guo and colleagues (Guo et al., 2021) who reported that SARS-CoV-2 infection of a microengineered gut-on-chip 3-D model mimicking the intestinal epithelium-vascular endothelium barrier by co-culture of Caco-2 cells, HT29 cells, and vascular endothelial cells under physiological fluid flow, showed permissiveness for viral infection, morphological changes with injury of intestinal villi, dispersed distribution of mucus-secreting cells, and reduced expression of E-cad, indicating the destruction of the intestinal barrier integrity. Using the mucin-secreting human colorectal adenocarcinoma HT29 cell line (Hanski et al., 1992; Niv et al., 1995), we also confirmed a significantly increased release of sE-cad from these cells after exposure to SARS-CoV-2. However there was a lack of detectable viral production from HT29 cells exposed to SARS-CoV-2. This lack of viral production was unlikely to be due to the low expression of ACE2 at the surface of these cells and/or the expression of membrane-associated mucins reported to exhibit potent anti-SARS-CoV-2 activity (Rebendenne et al., 2021), since we find detectable viral proteins inside the cells by western blot analysis. It may be due to the existence of an unknown intracellular restriction factor (e.g. ATP8B1 flippase mutation) (Rebendenne et al., 2021), that remains to be identified. This corroborate previous observations reported by our laboratory indicating that HT29 cells, in contrast to Caco-2 cells, are poorly susceptible to SARS-CoV-2 replication and do not sustain viral production (Wurtz et al., 2021). The SARS-CoV-2-induced CDH1/E-cad gene down-modulation and E-cad protein expression was also recently reported in two human mammary epithelial cell lines (MCF10A and MCF12A) transduced with the human ACE2 (Lai et al., 2021).

In addition to the dysregulation of E-cad expression, we also found that SARS-CoV-2 infection of Caco-2 cells affects the expression of molecules involved in tight junctions (JAM-A and occludin) and gap junctions (connexin), suggesting a drastic effect on most or all molecules intended to maintain the integrity of the intercellular junctions between intestinal epithelial cells. We also found that SARS-CoV-2 infection of Caco-2 cells leads to an increased expression of zonulin. Zonulin is overexpressed in tissues and sera of subjects with intestinal permeability (Tripathi et al., 2009; Fasano, 2011). Our data also corroborate the observations recently published by Lai and colleagues (Lai et al., 2021) who reported that SARS-CoV-2 infection of ACE2-transduced human mammary epithelial cell lines is associated to loss of epithelial markers (E-cadherin and ZO-1) and gain of mesenchymal markers (N-cadherin, vimentin, and fibronectin). These results support the hypothesis that in COVID-19 patients shedding SARS-CoV-2 in stools, the virus is likely able to destroy the intercellular junctions insured by E-cad and other cell adhesion molecules thereby creating epithelium micro-damage allowing the transmigration of pathogens. The cleavage of E-cad will also result in the deregulation of the antiviral immune response. If later proven *in vivo*, this hypothesis that considers early intestinal infectious foci, could explain why some COVID-19 patients may show intestinal disorders that precede lung disorders. Interestingly, Raghavan and colleagues (Raghavan et al., 2021) very recently reported that a recombinant SARS-CoV-2 spike protein S1 induces the degradation of junctional proteins (VE-cadherin, JAM-A, Connexin-43 and PECAM-1) that maintain endothelial barrier integrity. This result corroborates and strengthen our present data. Moreover, in severe COVID-19 patients the plasma levels of adhesion molecules such as intercellular adhesion molecule 1 (I-CAM-1), vascular cell adhesion molecule-1 (VCAM-1), vascular adhesion protein-1 (VAP-1), were reported to be elevated (Tong et al., 2020; Escher et al., 2020). In addition, it was recently reported SARS-CoV-2 E protein can interfere with control of cell polarity and cell-cell junction integrity in human epithelial cells by binding to the PALS1 PDZ domain (Javorsky et al., 2021). In addition, the integrity of the GIT epithelium is controlled by the homotypic interaction of E-cad between adjacent cells. It was also recently suggested that the SARS-CoV-2 ORF7b interfere with important cellular processes that involve leucine-zipper formation such as the transmembrane leucine zipper dependent dimerization of E-cad (Fogeron et al., 2021).

*CDH1* gene modulation (e.g., by methylation of the *CDH1* gene), aberrant splicing of the E-cad transcript (e.g., premature termination codon mutation), and E-cad degradation (e.g., by enhancing ADAM-10 sheddase activity), counts among the mechanisms used by several pathogens to achieve tissue invasion and transmigration through epithelial cell barriers (reviewed in Devaux et al., 2019). Although our results were obtained using a model of a GIT epithelial cell line (a human colorectal adenocarcinoma) rather than a polarized monolayer of human intestinal epithelial cells, they suggest that in COVID-19 patients the dissemination of SARS-CoV-2 could trigger damage to the intestinal epithelium barrier thereby favouring the spread of the virus to other tissues (e.g., pulmonary tissues). Moreover, a lower expression of E-cad at the site of infection, could interfere with the homing of immune cells and could trigger their rerouting far from the infection site (Reyat et al., 2017). The release of sE-cad could also serve as a decoy for diverting immune cells expressing E-cad, CD103 or KLRG1 from their function (e.g., KLRG1+ CD8+ T-cells subpopulation) after engagement of such receptors with sE-cad (Streeck et al., 2011). The modulation of E-cad expression on the host epithelial cells and sE-cad release could therefore be considered as a very efficient stratagem to prevent the immune system being activated against SARS-CoV-2. It may also contribute to triggering modulation in the diversity of bacterial species present in the GIT (Dhar and Mohanty, 2020; Gu S. et al., 2020; Gu J. et al., 2020; Zuo et al., 2020), this SARS-CoV-2-associated dysbiosis being responsible for the GIT syndrome observed in COVID-19 patients.

Recently we quantified the replication of different SARS-CoV-2 isolates namely B, B.1.416, B.1.367, B.1.1.7 (Alpha), B.1.351 (Beta), B.1.617.2 (Delta), P.1 (Gama), A.27 and B.1.160 in three different cell lines including Caco-2. Although the genotypes B.1.1.7 and B.1.351 (which are considered to be variants of concern, VOC), presented lower replication capacities in Calu-3 cells compared to the virus from lineages B or B.1.160, we found that the different viral isolates replicate similarly in Caco-2 cells (Pires de Souza et al., 2022). To enter susceptible cells, SARS-CoV-2 binds to ACE2. ACE2 initially is known to allow the conversion of angiotensin II (Ang II) into angiotensin-(1-7) hereafter Ang-(1-7) (reviewed in Devaux et al., 2020). Ang II could induce store-operated calcium entry (SOCE) (Guo et al., 2021). Under physiological conditions when the Ang II peptide is hydrolyzed by ACE2 into Ang-(1-7), the Ang-(1-7) binds to the MAS receptor (a G-protein-coupled receptor well known to be expressed in the kidney, adrenals and vascular system cells among others), leading to the inhibition of the PAK1/NF-KB/Snail 1 signaling pathway that can otherwise be activated by SOCE. In the absence of Ang-(1-7), this signaling cascade is activated and results in the inhibition of E-cad expression (Yu et al., 2016). In light of these data, we postulated that SARS-CoV-2-mediated reduction of ACE2 peptidase activity (either by reduction of ACE2 mRNA expression, or by blocking the peptidase function of ACE2), is very likely to be accompanied by a down-regulation of E-cad surface expression. In our model of Caco-2 cells, we tested the expression of gene coding for AT1R (the Ang II receptor), MAS1 and MA1-L (the Ang-(1-7) receptor) (**Supplementary Figure 1**). We found a detectable but very low expression of AT1R and MA1-L mRNA in Caco-2 cells, while MAS1 was not detected. In addition, our experiments were performed in the absence of Ang II and Ang-(1-7) adjunction to the cell culture medium, therefore if the PAK1/NF-KB/Snail 1 signaling pathway does play a role in the down regulation of E-cad by SARS-CoV-2 in Caco-2 cells, the regulation could be downstream of AT1R and MAS1/MA1-L. Yet, cell-surface molecules such as AT1R and ACE2 are interconnected. Recently, we reported evidence that engagement of AT1R with angiotensin II receptor blockers triggers higher cell-surface expression of ACE2 and enhance SARS-CoV-2 replication (Pires de Souza et al., 2021). Moreover, although we recently reported that ACE2 gene expression and ACE2 peptidase activity is reduced in COVID-19 patients with the accumulation of Ang II, their plasma concentrations of Ang-(1-7) remain stable, likely *via* Ang-(1-7) production through the alternative NEP/TOP pathway (Osman et al., 2021). This suggests that in COVID-19 patients, the Ang-(1-7)/MASR is expected to keep the PAK1/NF-KB/Snail 1 signaling pathway under inhibition. However, it was recently reported *in vitro* that SARS-CoV-2 spike induces breast cancer metastasis (epithelial mesenchymal transition) through activation of NF-KB/Snail and that knockdown of Snail by lentiviral-based shRNA (shSnail-1) leads to E-cad expression rescue (Lai et al., 2021).

In parallel with the Ang-(1-7)/MASR axis, it remains possible that the E-cad inhibition observed in SARS-CoV-2-infected Caco-2 can be completed either by signal transduction leading to *CDH1* gene down-regulation through ACE2 after SARS-CoV-2-binding to its cellular receptor, or by activation of sheddases able to cleave the membrane E-cad. E-cad is a transmembrane protein containing five extracellular repeated domains (EC1 to EC5), a transmembrane region, and an intracytoplasmic C-terminal region (CTR). The extracellular portion of E-cad forms junction with cell adhesion molecules on proximal cell, whereas the CTR binds β-catenin and other signaling molecules (reviewed in Devaux et al., 2019). Extracellular E-cad cleavage can be achieved by endoproteases (which cleave internal peptide bonds) belonging to the large family of sheddases (Grabowska and Day, 2012). The human sheddases include zinc-dependent matrix metalloproteases (matrilysin/MMP-2, 3, 7, 9, and 14) (Lee et al., 2007; Symowicz et al., 2007; Klein and Bischoff, 2011), and members of the α-disintegrin metalloproteases family (adamalysin/ADAM-10 and -15) (Maretzky et al., 2008; Najy et al., 2008; Giebeler and Zigrino, 2016), among others (reviewed in Grabowska and Day, 2012; Devaux et al., 2019). For instance, the eukaryotic ADAM-10 sheddase, abundantly expressed throughout the gastrointestinal tract and during normal intestinal homeostasis (reviewed in Dempsey, 2017), catalyzes a cleavage of E-cad that results in the release of the N-terminal sE-cad fragment and a C-terminal fragment (Maretzky et al., 2005). Another member of the ADAM family, the tumour necrosis factor α-convertase ADAM-17, was previously reported to be involved in soluble ACE2 shedding (Lambert et al., 2005; Zipeto et al., 2020). Unexpectedly, in our experiments we found a significantly lower expression of genes encoding the ADAM-10 and ADAM-17 sheddases in Caco-2 cells 24 hours after infected with SARS-CoV-2. It remains possible that these sheddases are activated during the early phase of infection to induce the release of sE-Cad and sACE2 following viral infection and then that a feedback regulation loop down-modulates the ADAM-10 and ADAM-17 genes expression. Interestingly, a few years ago Qian and colleagues (Qian et al., 2013) reported that over-expression of ACE2 attenuates the metastasis of cancer cells through inhibition of epithelial-mesenchymal transition, because it induces the up-regulation of cell surface E-cad. Accordingly, the down-regulation of *CDH1*/E-cad gene expression could be linked to the down-regulation of ACE2 induced by SARS-CoV-2 infection and be triggered through activation of the NF-KB/Snail pathway, as suggested by Lai and colleagues (Lai et al., 2021). The results obtained for the ADAM-10 gene expression should be considered preliminary observations and an exhaustive analysis of all the sheddases that may be involved in the release of sE-cad will have to be undertaken subsequently. Preliminary analyses indicate that SARS-CoV-2 infection induce the modulation of several other human sheddases (data not shown). In addition, in COVID-19 patients it is possible that the dysbiosis induced by SARS-CoV-2 infection results in the activation of prokaryotic sheddases (e.g., the HtrA bacterial serine protease), able to cleave E-cad. This hypothesis should be investigated, since it is likely to contribute to the induction of a pro-inflammatory process in the intestinal epithelium.

According to these data and in agreement with other publications (Lamers et al., 2020; Zhang et al., 2020b; Xiao et al., 2020a), we speculated that the gastrointestinal tract can be an alternative route for SARS-CoV-2 infection in humans. Zhang and colleagues suggested that although most virus would be dead in the acid environment in the stomach, it remains possible that the saliva and secretions could carry the virus into the digestive tract where viral replication may be sustained in epithelial cells. In K18-hACE2 transgenic mice expressing human ACE2 infected with SARS-CoV-2 *via* intranasal administration, extensive necrosis and inflammation of the lamina propria of intestinal villi associated to cell damages and increased intestinal permeability were observed. Recently, experiments performed in a nonhuman primate model support this hypothesis (Jiao et al., 2021). The intragastric inoculation of nonhuman primates with SARS-CoV-2 resulted in the productive infection of digestive tissues and inflammation in both the lung and digestive tissues. This route of inoculation induced Inflammatory cytokines production and immunohistochemistry and Alcian blue/periodic acid–Schiff staining showed decreased Ki67, increased cleaved caspase 3, and decreased numbers of mucin-containing goblet cells, suggesting that the inflammation process induced by intragastric SARS-CoV-2 impaired the gastrointestinal barrier. Moreover, ACE2 and TMPRSS molecules are highly expressed in the epithelial cells of the oral cavity and these cells are susceptible to SARS-CoV-2 infection and sustain viral replication (Huang et al., 2021). Altogether these observations support the hypothesis of a possible fecal-oral route of infection.

Taken as a whole, our results provide new insight into the complex molecular interactions between SARS-CoV-2 and intestinal cells and highlight the fact that the infection of Caco-2 cells is accompanied by down-modulation of the *CDH1*/E-cad gene, lower cell surface expression of E-cad and increased release of sE-cad. We speculate that similar changes in E-cad expression probably occur in the GIT of COVID-19 patients leading to micro-damage to the epithelium barrier, a deregulation of the immune response, pro-inflammation, and the invasion of distant host organs/tissues.

## Data Availability Statement

The original contributions presented in the study are included in the article/**Supplementary Material**. Further inquiries can be directed to the corresponding author.

## Ethics Statement

The animal study have been submitted to the Institutional Animal Care and Use Committee of CIPHE approved by French authorities (CETEA DSV -APAFIS#26484-2020062213431976 v6).

## Author Contributions

IO and CD contributed to the design of the study and conceived the manuscript. IO, GP, and DB performed the viral infections. IO and CG performed the *in vitro* experiments. J-PB and DB performed the electron microscopy analysis. AZ performed the *in vivo* experiments. HL performed the pathological examination. BM, J-LM, BL and CD supervised the work. CD wrote the first draft of the manuscript. All authors participated in correction of the manuscript. All authors contributed to the article and approved the submitted version.

## Funding

This work was supported by the French Government under the « Investissements d’avenir » (Investments for the Future) program managed by the Agence Nationale de la Recherche (French ANR, National Agency for Research, reference, Méditerranée Infection 10-IAHU-03 to Didier Raoult), annual funds from Aix-Marseille university and IRD to the MEPHI research unit, the COVIDHUMICE project (Fondation pour la Recherche Médicale-ANR Flash Covid-COVI-0066 to BM) and the PHENOMIN (French National Infrastructure for mouse Phenogenomics, ANR-10-INBS-07, to BM). Other funding sources were limited to the salaries of the authors (Centre National de la Recherche Scientifique for CD, Caisse Nationale de Sécurité Sociale de Djibouti for IO), with no other role or involvement.

## Conflict of Interest

The authors declare that the research was conducted in the absence of any commercial or financial relationships that could be construed as a potential conflict of interest.

## Publisher’s Note

All claims expressed in this article are solely those of the authors and do not necessarily represent those of their affiliated organizations, or those of the publisher, the editors and the reviewers. Any product that may be evaluated in this article, or claim that may be made by its manufacturer, is not guaranteed or endorsed by the publisher.

## Supplementary Material

Supplementary Figure 1 Relative expression of different cellular mRNA in virus-free Caco-2 cells, in particular the mRNA encoding SARS-CoV-2 receptor/coreceptors as well as AngII and Ang-(1-7) receptors, and mRNA encoding cell adhesion and tight junctions molecules. The results are expressed as RE where RE = 2^(−ΔCT)^.

Supplementary Figure 2
**(A)** Relative expression of different cellular mRNA in virus-free HT29 cells, in particular the mRNA encoding SARS-CoV-2 receptor/coreceptors as well as AngII and Ang-(1-7) receptors, and mRNA encoding cell adhesion molecules. The results are expressed as RE where RE = 2^(−ΔCT)^. **(B)** Confocal microscope analysis of E-cad, ACE2, occludin and actin expression on virus-free HT29 cells. The experiment was performed using cells permeabilized with 0.1% Triton X-100 (upper panel) and unpermeabilized cells (lower panel). **(C)** Quantification of the viral release from HT29 cell by qRT-PCR: ΔCT represents the CT value obtained subtracted from the CT value at time T=0 (CT - CT_0_), where T=0 is the moment immediately after removing the inoculum used in the adsorption step. Each value is the mean of triplicates. **(D)** Quantification of SARS-CoV-2 viral antigen in the HT29 cells exposed to the virus at an MOI of 0.5 for 4 hours, 24 hours and 48 hours, by antibody-detection of SARS-CoV-2 nucleocapsid protein using high-speed capillary electrophoresis JessTM automated immunoblotting system. Although the HT29 cells can be infected by SARS-CoV-2 (as shown in **D**), viral release was too low to be quantifiable in the culture supernatants of HT29 cells (as shown in **C**).

## References

[B1] Bouhaddou M. Memon D. Meyer B. White K. M. Rezelj V. V. Correa Marrero M.. (2020). The Global Phosphorylation Landscape of SARS-CoV-2 Infection. Cell 182 (3), 685–712. doi: 10.1016/j.cell.2020.06.034 32645325 PMC7321036

[B2] Brogna C. Cristoni S. Petrillo M. Bisaccia D. R. Lauritano F. Montan L.. (2022). The First Report on Detecting SARS-CoV-2 Inside Human Fecal-Oral Bacteria: A Case Series on Asymptomatic Family Members and a Child With COVID-19. F1000Research 11, 135. doi: 10.12688/f1000research.77421.1 PMC11502994 39464247

[B3] Camargo S. M. Singer D. Makrides V. Huggel K. Pos K. M. Wagner C. A.. (2009). Tissue-Specific Amino Acid Transporter Partners ACE2 and Collectrin Differentially Interact With Hartnup Mutations. Gastroenterology 136, 872–882. doi: 10.1053/j.gastro.2008.10.055 19185582 PMC7094282

[B4] Cantuti-Castelvetri L. Ojha R. Pedro L. D. Djannatian M. Franz J. Kuivanen S.. (2020). Neuropilin-1 Facilitates SARS-CoV-2 Cell Entry and Infectivity. Science 370, 856–860. doi: 10.1126/science.abd2985 33082293 PMC7857391

[B5] Chelakkot C. Ghim J. Ryu S. H. (2018). Mechanisms Regulating Intestinal Barrier Integrity and its Pathological Implications. Exp. Mol. Med. 50, 103. doi: 10.1038/s12276-018-0126-x PMC6095905 30115904

[B6] Choi W. I. Kim I. B. Park S. J. Ha E. H. Lee C. W. (2021). Comparison of the Clinical Characteristics and Mortality of Adults Infected With Human Coronaviruses 229E and OC43. Sci. Rep. 11 (1), 4499. doi: 10.1038/s41598-021-83987-3 33627764 PMC7904943

[B7] Chu H. Chan J. F. W. Yuen T. T. T. Shuai H. Yuan S. Wang Y.. (2020). Comparative Tropism, Replication Kinetics, and Cell Damage Profiling of SARS-CoV-2 and SARS-CoV With Implications for Clinical Manifestations, Transmissibility, and Laboratory Studies of COVID-19: An Observational Study. Lancet 1, e14–e23. doi: 10.1016/S2666-5247(20)30004-5 PMC7173822 32835326

[B8] Cohen T. (2001). Neuroendocrine Cells Along the Digestive Tract Express Neuropilin-2. Biochem. Biophys. Res. Commun. 284, 395–403. doi: 10.1006/bbrc.2001.4958 11394892

[B9] Collins A. R. (1990). Comparison of the Replication of Distinct Strains of Human Coronavirus OC43 in Organotypic Human Colon Cells (Caco-2) and Mouse Intestine. Adv. Exp. Med. Biol. 276, 497–503. doi: 10.1007/978-1-4684-5823-7_69 2103102

[B10] D'Costa Z. J. Jolly C. Androphy E. J. Mercer A. Matthews C. M. Hibma M. H. (2012). Transcriptional Repression of E-Cadherin by Human Papillomavirus Type 16 E6. PloS One 7 (11), e48954. doi: 10.1371/journal.pone.0048954 23189137 PMC3506579

[B11] Daly J. L. Simonetti B. Klein K. Chen K. E. Williamson M. K. Anton-Plagaro C.. (2020). Neuropilin-1 is a Host Factor for SARS-CoV-2 Infection. Science 370 (6518), 861–865. doi: 10.1126/science.abd3072 33082294 PMC7612957

[B12] D’Amico F. Baumgart D. C. Danese S. Peyrin-Biroulet L. (2020). Diarrhea During COVID-19 Infection: Pathogenesis, Epidemiology, Prevention and Management. Clin. Gastroenterol. Hepatol. 18, 1663–1672. doi: 10.1016/j.cgh.2020.04.001 32278065 PMC7141637

[B13] Dempsey P. J. (2017). Role of ADAM10 in Intestinal Crypt Homeostasis and Tumorigenesis. Biochem. Biophys. Acta 1864 (11), 2228–2239. doi: 10.1016/j.bbamcr.2017.07.011 PMC5632589 28739265

[B14] Dergham J. Delerce J. Bedotto M. La Scola B. Moal V. (2021). Isolation of Viable SARS-CoV-2 Virus From Feces of an Immunocompromised Patient Suggesting a Possible Fecal Mode of Transmission. J. Clin. Med. 10, 2696. doi: 10.3390/jcm10122696 34207314 PMC8235306

[B15] Devaux C. A. Lagier J.-C. Raoult D. (2021). New Insights Into the Physiopathology of COVID-19: SARS-CoV-2-Associated Gastrointestinal Illness. Front. Med. 8. doi: 10.3389/fmed.2021.640073 PMC7930624 33681266

[B16] Devaux C. A. Mezouar S. Mege J.-L. (2019). The E-Cadherin Cleavage Associated to Pathogenic Bacteria Infections Can Favor Bacterial Invasion and Transmigration, Dysregulation of the Immune Response and Cancer Induction in Humans. Front. Microbiol. 10. doi: 10.3389/fmicb.2019.02598 PMC6857109 31781079

[B17] Devaux C. A. Rolain J. M. Raoult D. (2020). ACE2 Receptor Polymorphism: Susceptibility to SARS-CoV-2, Hypertension, Multiorgan Failure, and COVID-19 Disease Outcome. J. Microbiol. Immunol. Infect. 53, 425–435. doi: 10.1016/j.micinf.2020.03.003 32414646 PMC7201239

[B18] Dhar D. Mohanty A. (2020). Gut Microbiota and COVID-19 Possible Link and Implications. Virus Res. 285, 198018. doi: 10.1016/j.virusres.2020.198018 32430279 PMC7217790

[B19] Edouard S. Jaafar R. Orain N. Parola P. Colson P. la Scola B.. (2021). Automated Western Immunoblotting Detection of Anti-SARS-CoV-2 Serum Antibodies. Eur. J. Clin. Microbiol. Infect. Dis. 40 (6), 1309–1317. doi: 10.1007/s10096-021-04203-8 33660134 PMC7928199

[B20] Escher R. Breakey N. Lammle B. (2020). Severe COVID-19 Infection Associated With Endothelial Activation. Thromb. Res. 190, 62. doi: 10.1016/j.thromres.2020.04.014 32305740 PMC7156948

[B21] Fairweather S. J. Broer A. O’Mara M. L. Broer S. (2012). Intestinal Peptidases From Functional Complexes With Neutral Amino Acid Transporter B0AT1. Biochem. J. 446, 135–148. doi: 10.1042/BJ20120307 22677001 PMC3408045

[B22] Fang D. Ma J. Guan J. (2020). Manifestation of Digestive System in Hospitalized Patients With Novel Coronavirus Pneumonia in Wuhan, China: A Single-Center, Descriptive Study. Chin. J. Dig. 40, E005. doi: 10.3760/cma.j.i.ssn.0254-1432.2020.0005

[B23] Fasano A. (2011). Zonulin and Its Regulation of Intestinal Barrier Function: The Biological Door to Inflammation, Autoimmunity, and Cancer. Physiol. Rev. 91, 151–175. doi: 10.1152/physrev.00003.2008 21248165

[B24] Fogeron M. L. Montserret R. Zehnder J. Nguyen M. H. Dujardin M. Brigandat L.. (2021). SARS-CoV-2 ORF7b: Is a Bat Virus Protein Homologue a Major Cause of COVID-19 Symptoms? bioRxiv. doi: 10.1101/2021.02.05.428650

[B25] Giebeler N. Zigrino P. (2016). A Disintegrin and Metalloprotease (ADAM): Historical Overview of Their Functions. Toxins 8, 122. doi: 10.3390/toxins8040122 27120619 PMC4848645

[B26] Grabowska M. M. Day M. L. (2012). Soluble E-Cadherin: More Than a Symptom of Disease. Front. Biosci. 17, 1948–1964. doi: 10.2741/4031 PMC4183062 22201848

[B27] Gu S. Chen Y. Wu Z. Chen Y. Gao H. Lv L.. (2020). Alterations of the Gut Microbiota in Patients With Coronavirus Disease 2019 or H1N1 Influenza. Clin. Infect. Dis. 71, 2669–2678. doi: 10.1093/cid/ciaa709 32497191 PMC7314193

[B28] Gu J. Han B. Wang J. (2020). COVID-19: Gastrointestinal Manifestations and Potential Fecal-Oral Transmission. Gastroenterology 158, 1518–1519. doi: 10.1053/j.gastro.2020.02.054 32142785 PMC7130192

[B29] Guo Y. Luo R. Wang Y. Deng P. Song T. Zhang M.. (2021). SARS-CoV-2 Induced Intestinal Responses With a Biomimetic Human Gut-on-Chip. Sci. Bull. 66, 783–793. doi: 10.1016/j.scib.2020.11.015 PMC7704334 33282445

[B30] Guo R. W. Yang L. X. Li M. Q. Pan X. H. Liu B. Deng Y. L. (2012). Stim1- and Orai1- Mediated Store-Operated Calcium Entry is Critical for Angiotensin II-Induced Vascular Smooth Muscle Cell Proliferation, Cardiovasc. Res 93, 360–370. doi: 10.1093/cvr/cvr307 22108917

[B31] Hamming I. Timens W. Bulthuis M. Lely T. Navis G. van Goor H. (2004). Tissue Distribution of ACE2 Protein, the Functional Receptor for SARS Coronavirus. J. Pathol. 203, 631–637. doi: 10.1002/path.1570 15141377 PMC7167720

[B32] Han C. Duan C. Zhang S. Spiegel B. Shi H. Wang W.. (2020). Digestive Symptoms in COVID-19 Patients With Mild Disease Severity: Clinical Presentation, Stool Viral RNA Testing, and Outcomes. Am. J. Gastroenterol. 115, 916–923. doi: 10.14309/ajg0000000000000664 32301761 PMC7172493

[B33] Hansel D. E. Wilentz R. E. Yeo C. J. Schulick R. D. Montgomery E. Maitra A. (2004). Expression of Neuropilin-1 in High-Grade Dysplasia, Invasive Cancer, and Metastases of the Human Gastrointestinal Tract. Am. J. Surg. Pathol. 28, 347–356. doi: 10.1097/00000478-200403000-00007 15104297

[B34] Hanski C. Stolze B. Riecken E. G. (1992). Tumorigenicity, Mucin Production and AM-3 Epitope Expression in Clones Selected From the HT-29 Colon Carcinoma Cell Line. Int. J. Cancer 50 (6), 924–929. doi: 10.1002/ijc.2910500618 1372882

[B35] Haspel N. Zanuy D. Nussinov R. Teesalu T. Ruoslahti E. Aleman C. (2011). Binding of a Peptide to Neuropilin-1 Receptor: A Molecular Modeling Approach. Biochemistry 50 (10), 1755–1762. doi: 10.1021/bi101662j PMC3051018 21247217

[B36] Heneghan C. Spencer E. Brassey J. Plüddemann A. Onakpoya I. J. Evans D. H.. (2021). SARS-CoV-2 and the Role of Orofecal Transmission: Systematic Review. F1000 Res. 10, 232. doi: 10.12688/f1000research.51592.1 PMC8749895 35035883

[B37] Hoffmann N. Kleine-Weber H. Schroeder S. Krüger N. Herrler T. Erichsen S.. (2020). SARS-CoV-2 Cell Entry Depends on ACE2 and TMPRSS2 and Is Blocked by a Clinically Proven Protease Inhibitor. Cell. 181, 1–10. doi: 10.1016/j.cell.2020.02.052 32142651 PMC7102627

[B38] Huang N. Pérez P. Kato T. Mikami Y. Okuda K. Gilmore R. C.. (2021). SARS-CoV-2 Infection of the Oral Cavity and Saliva. Nat. Med. 27 (5), 892–903. doi: 10.1038/s41591-021-01296-8 33767405 PMC8240394

[B39] Huang C. Wang Y. Li X. Ren L. Zhao J. Hu Y. (2020). Clinical Features of Patients Infected With 2019 Novel Coronavirus in Wuhan, China. Lancet 395, 497–506. doi: 10.1016/S0140-6736(20)30183-5 31986264 PMC7159299

[B40] Javorsky A. Humbert P. O. Kvansakul M. (2021). Structural Basis of Coronavirus E Protein Interactions With Human PALS1 PDZ Domain. Commun. Biol. 4, 724. doi: 10.1038/s42003-021-02250-7 34117354 PMC8196010

[B41] Jiao L. Li H. Xu J. Yang M. Ma C. Li J.. (2021). The Gastrointestinal Tract is an Alternative Route for SARS-CoV-2 Infection in a Nonhuman Primate. Gastroenterology 160 (5), 1647–1661. doi: 10.1053/j.gastro.2020.12.001 33307034 PMC7725054

[B42] Klein T. Bischoff R. (2011). Physiology and Pathophysiology of Matrix Metalloproteases. Amino Acids 41, 271–290. doi: 10.1007/s00726-010-0689-x 20640864 PMC3102199

[B43] Lai Y. J. Chao C. H. Liao C. C. Lee T. A. Hsu J. M. Chaou W. C.. (2021). Epithelial-Mesenchymal Transition Induced by SARS-CoV-2 Required Transcriptional Upregulation of Snail. Am. J. Cancer Res. 11 (5), 2278–2290. 34094684 PMC8167694

[B44] Lambert D. W. Yarski M. Warner F. J. Thornhill P. Parkin E. T. Smith A. I.. (2005). Tumor Necrosis Factor-α Convertase (ADAM17) Mediates Regulated Ectodomain Shedding of the Severe-Acute Respiratory Syndrome-Coronavirus (SARS-CoV) Receptor, Angiotensin-Converting Enzyme-2 (Ace2). J. Biol. Chem. 280 (34), 30113–30119. doi: 10.1074/jbc.M505111200 15983030 PMC8062222

[B45] Lamers M. M. Beumer J. van der Vaart J. Knoops K. Puschhof J. Breugem T. I.. (2020). SARS-CoV-2 Productively Infects Human Gut Enterocytes. Science 369, 50–54. doi: 10.1126/science.abc1669 32358202 PMC7199907

[B46] Le Bideau M. Wurtz N. Baudoin J. P. La Scola B. (2021). Innovative Approach to Fast Electron Microscopy Using the Example of a Culture of Virus-Infected Cells: An Application to SARS-CoV-2. Microorganisms 9 (6), 1194. doi: 10.3390/microorganisms9061194 34073053 PMC8228702

[B47] Lee K. H. Choi E. Y. Hyun M. S. Jang B. I. Kim T. N. Kim S. W.. (2007). Association of Extracellular Cleavage of E-Cadherin Mediated by MMP-7 With HGF-Induced *In Vitro* Invasion in Human Stomach Cancer Cells. Eur. Surg. Res. 39, 208–215. doi: 10.1159/000101452 17396032

[B48] Lee J. O. Kwun H. J. Jung J. K. Choi K. H. Min D. S. Jang K. L. (2005). Hepatitis B Virus X Protein Represses E-Cadherin Expression *via* Activation of DNA Methyltransferase 1. Oncogene 24, 6617–6625. doi: 10.1038/sj.onc.1208827 16007161

[B49] Letko M. Marzi A. Munster V. (2020). Functional Assessment of Cell Entry and Receptor Usage for SARS-CoV-2 and Other Lineage B Betacoronaviruses. Nat. Microbiol. 5, 562–569. doi: 10.1038/s41586-020-2012-7 32094589 PMC7095430

[B50] Li X. Y. Dai W. J. Wu S. N. Yang X. Z. Wang H. G. (2020). The Occurrence of Diarrhea in COVID-19 Patients. Clin. Res. Hepatol. Gastroenterol. 44, 284–285. doi: 10.1016/j.clinre.2020.03.017 32253163 PMC7270575

[B51] Lin L. Jiang X. Zhang Z. Huang S. Zhang Z. Fang Z.. (2020). Gastrointestinal Symptoms of 95 Cases With SARS-CoV-2 Infection. Gut 69, 997–1001. doi: 10.1136/gutjnl-2020-321013 32241899

[B52] Li Q. Sodroski C. Lowey B. Schweitzer C. J. Cha H. Zhang F.. (2016). Hepatitis C Virus Depends on E-Cadherin as an Entry Factor and Regulates its Expression in Epithelial-Tomesenchymal Transition. Proc. Natl. Acad. Sci. U.S.A. 113 (27), 7620–7625. doi: 10.1073/pnas.1602701113 27298373 PMC4941495

[B53] Maretzky T. Reiss K. Ludwig A. Buchholz J. Scholz F. Proksch E.. (2005). ADAM10 Mediates E-Cadherin Shedding and Regulates Epithelial Cell-Cell Adhesion, Migration, and β-Catenin Translocation. Proc. Natl. Acad. Sci. U.S.A. 102 (26), 9182–9187. doi: 10.1073/pnas.0500918102 15958533 PMC1166595

[B54] Maretzky T. Scholz F. Köten B. Proksch E. Saftig P. Reiss K. (2008). ADAM10-Mediated E-Cadherin Release is Regulated by Proinflammatory Cytokines and Modulates Keratinocyte Cohesion in Eczematous Dermatitis. J. Invest. Dermatol. 128, 1737–1746. doi: 10.1038/sj.jid.5701242 18200054

[B55] Meenan J. Mevissen M. Monajemi H. Radema S. A. Soule H. R. Moyle M.. (1996). Mechanisms Underlying Neutrophil Adhesion to Apical Epithelial Membranes. Gut 38 (2), 201–205. doi: 10.1136/gut.38.2.201 8801197 PMC1383023

[B56] Meng X. J. Liang T. J. (2021). SARS-CoV-2 Infection in the Gastrointestinal Tract: Fecal–Oral Route of Transmission for COVID-19? Gastroenterology 160, 1467–1474. doi: 10.1053/j.gastro.2021.01.00 33422479 PMC7790455

[B57] Najy A. J. Day K. C. Day M. L. (2008). The Ectodomain Shedding of E-Cadherin by ADAM15 Supports ErbB Receptor Activation. J. Biol. Chem. 283, 18393–18401. doi: 10.1074/jbc.M801329200 18434311 PMC2440598

[B58] Navabi N. McGuckin M. A. Lindén S. K. (2013). Gastrointestinal Cell Lines Form Polarized Epithelia With an Adherent Mucus Layer When Cultured in Semi-Wet Interfaces With Mechanical Stimulation. PloS One 8 (7), e68761. doi: 10.1371/journal.pone.0068761 23869232 PMC3712011

[B59] Niv Y. Schwartz B. Amsalem Y. Lamprecht S. A. (1995). Human HT-29 Colon Carcinoma Cells: Mucin Production and Tumorigenicity in Relation to Growth Phases. Anticancer Res. 15 (5B), 2023–2027. 8572596

[B60] Osman I. O. Melenotte C. Brouqui P. Million M. Lagier J.-C. Parola P.. (2021). Expression of ACE2, Soluble ACE2, Angiotensin I, Angiotensin II and Angiotensin-(1-7) Is Modulated in COVID-19 Patients. Front. Immunol. 12. doi: 10.3389/fimmu.2021.625732 PMC8236950 34194422

[B61] Owczarek K. Szczepanski A. Milewska A. Baster Z. Rajfur Z. Sarna M.. (2018). Early Events During Human Coronavirus OC43 Entry to the Cell. Sci. Rep. 8, 7124. doi: 10.1038/s41598-018-25640-0 29740099 PMC5940804

[B62] Pan L. M. Yang M. Sun P. Wang Y. Yan R. Li J.. (2020). Clinical Characteristics of COVID-19 Patients With Digestive Symptoms in Hubei, China: A Descriptive, Cross-Sectional, Multicenter Study. Am. J. Gastroenterol. 115, 766–773. doi: 10.14309/ajg.0000000000000620 32287140 PMC7172492

[B63] Pires de Souza G. A. Le Bideau M. Boschi C. Ferreira L. Wurtz N. Devaux C.. (2022). Emerging SARS-CoV-2 Genotypes Show Different Replication Patterns in Human Pulmonary and Intestinal Epithelial Cells. Viruses 14, 23. doi: 10.3390/v14010023 PMC8777977 35062227

[B64] Pires de Souza G. A. Osman I. O. Le Bideau M. Baudoin J.-P. Jaafar R. Devaux C.. (2021). Angiotensin II Receptor Blockers (ARBs Antihypertensive Agents) Increase Replication of SARS-CoV-2 in Vero E6 Cells. Front. Cell. Infect. Microbiol. 11. doi: 10.3389/fcimb.2021.639177 PMC8231006 34178717

[B65] Qi F. Qian S. Zhang S. Zhang Z.. (2020). Single Cell RNA Sequencing of 13 Human Tissues Identify Cell Types and Receptors of Human Coronaviruses. Biochem Biophys Res Commun 526, 135–140. doi: 10.1016/j.bbrc.2020.03.044 32199615 PMC7156119

[B66] Qian Y. R. Guo Y. Wan H. Y. Fan L. Feng Y. Ni L.. (2013). Angiotensin-Converting Enzyme 2 Attenuates the Metastasis of non-Small Cell Lung Cancer Through Inhibition of Epithelial-Mesenchymal Transition. Oncol. Rep. . 29, 2408–2414. doi: 10.3892/or.2013.2370 23545945

[B67] Raghavan S. Kenchappa D. B. Leo M. D. (2021). SARS-CoV-2 Spike Protein Induces Degradation of Junctional Proteins That Maintain Endothelial Barrier Integrity. Front. Cardiovasc. Med. 8. doi: 10.3389/fcvm.2021.687783 PMC8225996 34179146

[B68] Ramakrishnan M. A. (2016). Determination of 50% Endpoint Titer Using a Simple Formula. World J. Virol. 5 (2), 85–86. doi: 10.5501/wjv.v5.i2.85 27175354 PMC4861875

[B69] Rebendenne A. Priyanka R. Bonaventure B. Chaves Valadao A. L. Desmarets L. Rouillé Y.. (2021). Bidirectional Genome-Wide CRISPR Screens Reveal Host Factors Regulating SARS-CoV-2, MERS-CoV and Seasonal Coronaviruses. bioRxiv. doi: 10.1101/2021.05.19.444823 PMC11627114 35879413

[B70] Redd W. D. Zhou C. Hathorn K. E. McCarthy T. R. Bazarbashi A. N. Thompson C. C.. (2020). Prevalence and Characteristics of Gastrointestinal Symptoms in Patients With SARS-CoV-2 Infection in the United States: A Multicenter Cohort Study. Gastroenterology 159, 765–767. doi: 10.1053/j.gastro.2020.04.045 32333911 PMC7195377

[B71] Reyat J. S. Chimen M. Noy P. J. Szyroka J. Rainger G. Tomlinson M. G. (2017). ADAM10-Interacting Tetraspanins Tspan5 and Tspan17 Regulate VE-Cadherin Expression and Promote T Lymphocyte Transmigration. J. Immunol. 199, 666–676. doi: 10.4049/jimmunol.1600713 28600292 PMC5502317

[B72] Song Y. Liu P. Shi X. L. Chu Y. L. Zhang J. Xia J.. (2020). SARS-CoV-2 Induced Diarrhoea as Onset Symptom in Patient With COVID-19. Gut 69, 1143–1144. doi: 10.1136/gutjnl-2020-320891 32139552

[B73] Streeck H. Kwon D. S. Pyo A. Flanders M. Chevalier M. F. Law K.. (2011). Epithelial Adhesion Molecules can Inhibit HIV-1-Specific CD8+ T-Cell Functions. Blood 117, 5112–5122. doi: 10.1182/blood-2010-12-321588 21403126 PMC3109536

[B74] Sun S. H. Chen Q. Gu H. J. Yang G. Wang Y. X. Huang X. Y.. (2020). A Mouse Model of SARS-CoV-2 Infection and Pathogenesis. Cell Host Microbe 28, 124–133. doi: 10.1016/j.chom.2020.05.02 32485164 PMC7250783

[B75] Symowicz J. Adley B. P. Gleason K. J. Johnson J. J. Ghosh S. Fishman D. A.. (2007). Engagement of Collagen-Binding Integrins Promotes Matrix Metalloproteinase-9–Dependent E-Lycadherin Ectodomain Shedding in Ovarian Carcinoma Cells. Cancer Res. 67, 2030–2039. doi: 10.1158/0008-5472.CAN-06-2808 17332331

[B76] Teesalu T. Sugahara K. N. Kotamraju V. R. Ruoslahti E. (2009). C-End Rule Peptides Mediate Neuropilin-1-Dependent Cell, Vascular, and Tissue Penetration. Proc. Natl. Acd. Sci. U.S.A. 106 (38), 16157–16162. doi: 10.1073/pnas.0908201106 PMC2752543 19805273

[B77] Tong M. Jiang Y. Xia D. Xiong Y. Zheng Q. Chen F.. (2020). Elevated Serum Endothelial Cell Adhesion Molecules Expression in COVID-19 Patients. J. Infect. Dis. 222 (6), 894–898. doi: 10.1093/infdis/jiaa349 32582936 PMC7337874

[B78] Tripathi A. Lammers K. M. Goldblum S. Shea-Donohue T. Netzel-Arnett S. Buzza M. S.. (2009). Identification of Human Zonulin, a Physiological Modulator of Tight Junctions, as Prehaptoglobin-2. Proc. Natl. Acad. Sci. U.S.A. 106 (39), 16799–16804. doi: 10.1073/pnas.0906773106 19805376 PMC2744629

[B79] Vuille-Dit-Bille R. N. Camargo S. M. Emmenegger L. Sasse T. Kummer E. Jando J.. (2015). Human Intestine Luminal ACE2 and Amino Acid Transporter Expression Increased by ACE-Inhibitors. Amino Acids 47, 693–705. doi: 10.1007/s00726-014-1889-6 25534429

[B80] Wang D. Hu B. Hu C. Zhu F. Liu X. Zhang J.. (2020). Clinical Characteristics of 138 Hospitalized Patients With 2019 Novel Coronavirus-Infected Pneumonia in Wuhan, China. Jama 323, 1061–1069. doi: 10.1001/jama.2020.1585 32031570 PMC7042881

[B81] Wendling J. M. Saulnier A. Sabatier J. M. (2021). Share Food, Meals and Drinks: 10 Arguments Suggesting an Oral Transmission Route of COVID-19. Infect. Disorders-Drug Targets, 21. doi: 10.2174/1871526521666210716110603 34279208

[B82] Wölfel R. Corman V. M. Guggemos W. Seilmaier M. Zange S. Müller M. A.. (2020). Virological Assessment of Hospitalized Patients With COVID-19. Nature 581, 465–469. doi: 10.1038/s41586-020-2196-x 32235945

[B83] Wurtz N. Penant G. Jardot P. Duclos N. La Scola B. (2021). Culture of SARS-CoV-2 in a Panel of Laboratory Cell Lines, Permissivity, and Differences in Growth Profile. Eur. J. Clin. Microbiol. Infect. Dis. 40 (3), 477–484. doi: 10.1007/s10096-020-04106-0 33389257 PMC7778494

[B84] Xiao F. Sun J. Xu Y. Li F. Huang X. Li H.. (2020b). Infectious SARS-CoV-2 in Feces of Patient With Severe COVID-19. Emerg. Infect. Dis. 26, 1920–1922. doi: 10.3201/eid2608.200681 32421494 PMC7392466

[B85] Xiao F. Tang M. Zheng X. Liu Y. Li X. Shan H. (2020a). Evidence for Gastrointestinal Infection of SARS-CoV-2. Gastroenterology 158, 1831–1833. doi: 10.1053/j.gastro.2020.02.055 32142773 PMC7130181

[B86] Xu Y. Li X. Zhu B. Liang H. Fang C. Gong Y.. (2020). Characteristics of Pediatric SARS-CoV-2 Infection and Potential Evidence for Persistent Fecal Viral Shedding. Nat. Med. 26, 502–505. doi: 10.1038/s41591-020-0817-4 32284613 PMC7095102

[B87] Yan R. Zhang Y. Li Y. Xia L. Guo Y. Zhou Q. (2020). Structural Basis for the Recognition of the SARS-CoV-2 by Full-Length Human ACE2. Science 367, 1444–1448. doi: 10.1126/science.abb2762 32132184 PMC7164635

[B88] Yu D. C. Bury J. P. Tiernan J. Waby J. S. Staton C. A. Corfe B. M. (2011). Short-Chain Fatty Acid Level and Field Cancerization Show Opposing Associations With Enteroendocrine Cell Number and Neuropilin Expression in Patients With Colorectal Adenoma. Mol. Cancer 10, 27. doi: 10.1186/1476-4598-10-2 21401950 PMC3068125

[B89] Yu C. Tang W. Wang Y. Shen Q. Wang B. Cai C.. (2016). Downregulation of ACE2/Ang-(1–7)/Mas Axis Promotes Breast Cancer Metastasis by Enhancing Store-Operated Calcium Entry. Cancer Lett. 376 (2), 268–277. doi: 10.1016/j.canlet.2016.04.006 27063099

[B90] Zang R. Gomez Castro M. F. McCune B. T. Zeng Q. Rothlauf P. W. Sonnek N. M.. (2020). TMPRSS2 and TMPRSS4 Promote SARS-CoV-2 Infection of Human Small Intestinal Enterocytes. Sci. Immunol. 5, eabc3582. doi: 10.1126/sciimmunol.abc3582 32404436 PMC7285829

[B91] Zhang H. Kang Z. Gong H. Xu D. Wang J. Li Z.. (2020b). Digestive System is a Potential Route of COVID-19: An Analysis of Single-Cell Coexpression Pattern of Key Proteins in Viral Entry Process. Gut 69, 1010–1018. doi: 10.1136/gutjnl-2020-320953

[B92] Zhang H. Li H. B. Lyu J. R. Lei X. M. Li W. Wu G.. (2020a). Specific ACE2 Expression in Small Intestinal Enterocytes may Cause Gastrointestinal Symptoms and Injury After 2019-Ncov Infection. Int. J. Infect. Dis. 96, 19–24. doi: 10.1016/j.ijid.2020.04.027 32311451 PMC7165079

[B93] Zheng S. Fan J. Yu F. Feng B. Lou B. Zou Q.. (2020). Viral Load Dynamics and Disease Severity in Patients Infected With SARS-CoV-2 in Zhejiang Province, China, January–March 2020: Retrospective Cohort Study. BMJ 369, m1443. doi: 10.1136/bmj.m1443 32317267 PMC7190077

[B94] Zhu N. Zhang D. Wang W. Li X. Yang B. Song J. (2020). A Novel Coronavirus From Patients With Pneumonia in China 2019. N. Engl. J. Med. 382, 727–733. doi: 10.1056/NEJMoa2001017 31978945 PMC7092803

[B95] Zipeto D. Palmeira J. Argañaraz G. A. Argañaraz E. R. (2020). ACE2/ ADAM17/TMPRSS2 Interplay May Be the Main Risk Factor for COVID-19. Front. Immunol. 11. doi: 10.3389/fimmu.2020.576745 PMC7575774 33117379

[B96] Zuo T. Zhang F. Lui G. C. Y. Yeoh Y. K. Li A. Y. L. Zhan H.. (2020). Alterations in Gut Microbiota of Patients With COVID-19 During Time of Hospitalization. Gastroenterology 159, 944–955. doi: 10.1053/j.gastro.2020.05.048 32442562 PMC7237927

