# Supplementary figures and images for: Control of CDH1/E-Cadherin Gene Expression and Release of a Soluble Form of E-Cadherin in SARS-CoV-2 Infected Caco-2 Intestinal Cells: Physiopathological Consequences for the Intestinal Forms of COVID-19
Source: Front Cell Infect Microbiol. 2022 May 4;12:798767. doi: 10.3389/fcimb.2022.798767 (PMC9114883; doi:10.3389/fcimb.2022.798767)

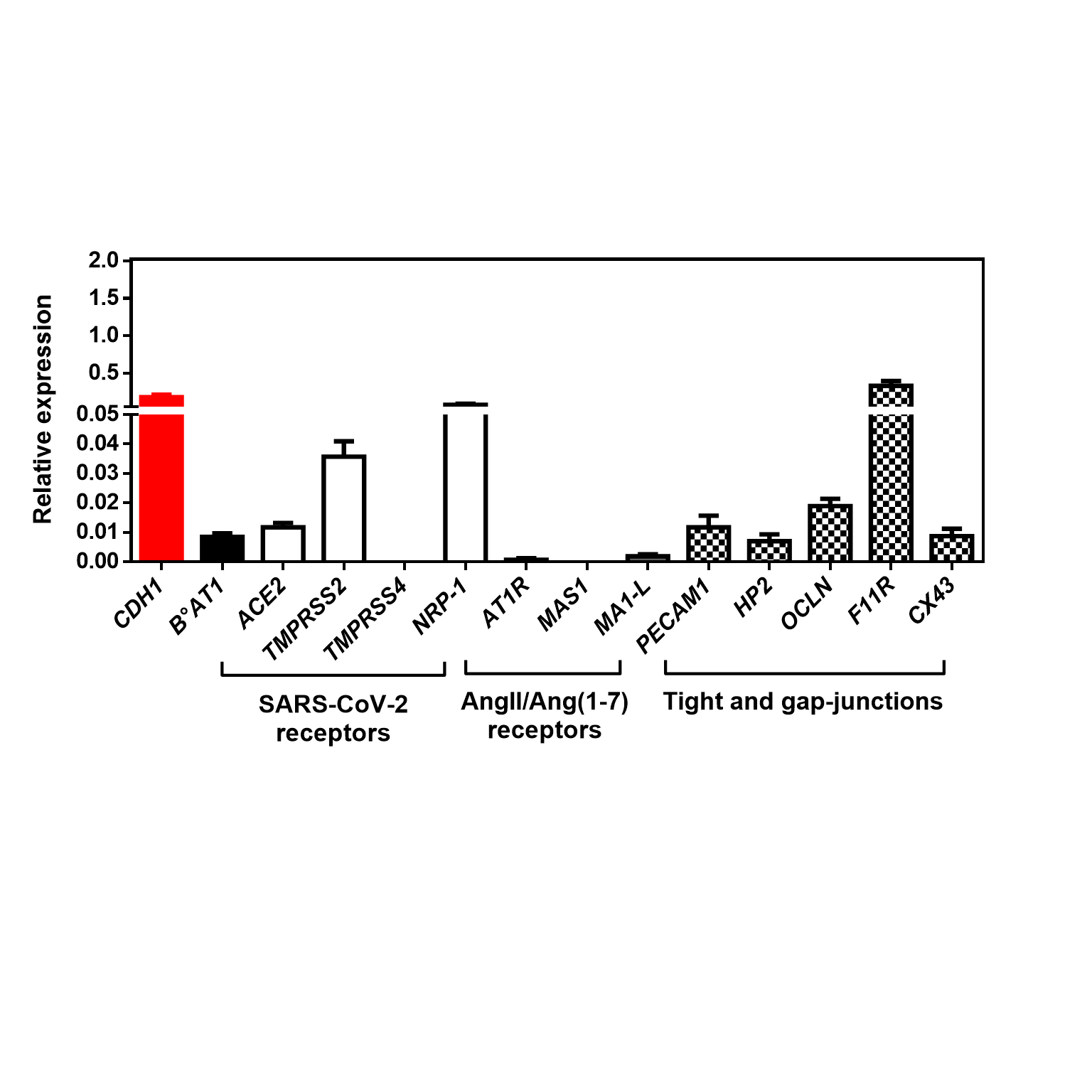

Supplement: Supplementary Figure 1 — Relative expression of different cellular mRNA in virus-free Caco-2 cells, in particular the mRNA encoding SARS-CoV-2 receptor/coreceptors as well as AngII and Ang-(1-7) receptors, and mRNA encoding cell adhesion and tight junctions molecules. The results are expressed as RE where RE = 2(−ΔCT). [file Image_1.tif]

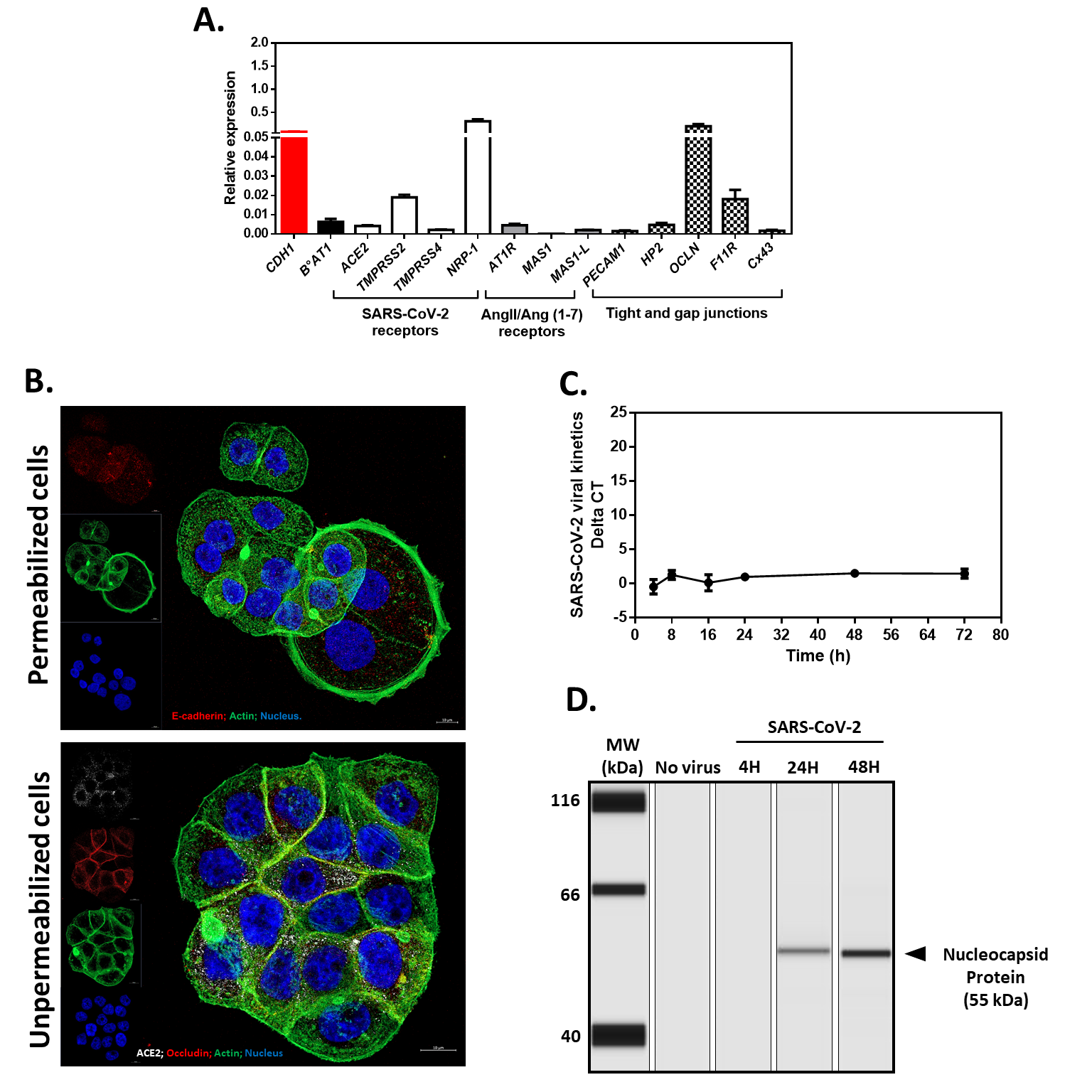

Supplement: Supplementary Figure 2 — (A) Relative expression of different cellular mRNA in virus-free HT29 cells, in particular the mRNA encoding SARS-CoV-2 receptor/coreceptors as well as AngII and Ang-(1-7) receptors, and mRNA encoding cell adhesion molecules. The results are expressed as RE where RE = 2(−ΔCT). (B) Confocal microscope analysis of E-cad, ACE2, occludin and actin expression on virus-free HT29 cells. The experiment was performed using cells permeabilized with 0.1% Triton X-100 (upper panel) and unpermeabilized cells (lower panel). (C) Quantification of the viral release from HT29 cell by qRT-PCR: ΔCT represents the CT value obtained subtracted from the CT value at time T=0 (CT - CT0), where T=0 is the moment immediately after removing the inoculum used in the adsorption step. Each value is the mean of triplicates. (D) Quantification of SARS-CoV-2 viral antigen in the HT29 cells exposed to the virus at an MOI of 0.5 for 4 hours, 24 hours and 48 hours, by antibody-detection of SARS-CoV-2 nucleocapsid protein using high-speed capillary electrophoresis JessTM automated immunoblotting system. Although the HT29 cells can be infected by SARS-CoV-2 (as shown in D), viral release was too low to be quantifiable in the culture supernatants of HT29 cells (as shown in C). [file Image_2.tif]
